# Deep tissue sensing of chiral molecules using polarization-enhanced photoacoustics

**DOI:** 10.1126/sciadv.ado8012

**Published:** 2025-03-19

**Authors:** Swathi Padmanabhan, Jaya Prakash

**Affiliations:** Department of Instrumentation and Applied Physics, Indian Institute of Science, Bengaluru 560012, India.

## Abstract

Chiral molecule sensing is typically performed using techniques like chromatography, electrophoresis, enzymatic assays, mass spectrometry, and chiroptical methods. While polarimetry allows for in vivo sensing up to 1 mm depth using ultraviolet-visible light, it is limited by dominant light scattering beyond this depth. We propose that photoacoustic sensing in the near-infrared II (NIR-II) window can enable deep tissue sensing as acoustic waves scatter less than light. To achieve this, we developed a photoacoustic polarization–enhanced optical rotation sensing (PAPEORS) system, capable of estimating optical rotation from photoacoustic signals and correlating it with chiral molecular concentration for depths up to 3.5 mm. Experiments were conducted using aqueous glucose solutions, naproxen, serum-based glucose samples, and ex vivo chicken tissue. PAPEORS achieved a detection limit of 80 mg/dl while using circularly polarized light with serum samples, demonstrating the potential for deep-tissue chiral molecular sensing. PAPEORS holds promise for in vivo sensing and easy miniaturization using single wavelength.

## Teaser

PAPEORS offers deep-tissue sensing of chiral biomolecules from optical rotation estimated using photoacoustic measurements.

## INTRODUCTION

Chirality is abundant in nature and plays a vital role in determining distinct properties of various organic and inorganic substances. Optically active molecules exhibit distinct properties compared to their enantiomeric counterparts (*1*, *2*). The plane of polarization of the transmitted light is rotated due to the light interaction with the optically active molecule, while with the polarization, properties change with scatterers in the medium. Many molecules present in the human body (proteins, amino acids, various sugars, and lactates) have a chiral center, which enables them to rotate the plane of the incident polarized light. Inorganic molecules, mostly drugs like ketamine (anesthesia drugs), steroids, and β-blockers (to treat cardiovascular problems), were found to be chiral in nature (*3*–*5*). The utility of optical rotation has been demonstrated by various optical techniques (*6*, *7*) to either analyze the rotation of the molecules or for detection/estimation. Thalidomide is a drug used for treating morning sickness during pregnancy. Notably, administration of incorrect enantiomeric counterpart [S(+) thalidomide] resulted in severe birth defects, while R(−) thalidomide was found to be safe and used as a sedative (*8*, *9*); this exemplified the importance of studying the effects of chiral molecules. Consequently, pharmaceutical drugs continue to undergo scrutiny before being administered, and drugs are analyzed in a neutralized (racemic) form or in a specific enantiomeric form to mitigate adverse effects.

Conventional methods for sensing chirality include chromatography, electrophoresis, and mass spectrometry (*10*). These are analytical detection methods and were calibrated for analyzing the enantiomeric excess in a sample and for the estimation of chiral molecular concentrations. Note that all these methods rely on analyzing body fluids like blood, saliva, or urine, with less scope for in vivo development. Chiroptical techniques considers the optical properties of chiral molecules like circular dichroism (CD), Raman optical activity, and optical rotary dispersion coupled with methods like spectroscopy (*11*, *12*), and polarimetry (*13*–*15*). A summary of the conventional and chiroptical methods highlighting various parameters and their feasibility in biosensing are shown in tables S1 and S2. Currently, available chiroptical techniques pose many challenges for in vivo characterization and quantification (*10*, *11*, *16*).

Polarimetry (*17*–*19*) was explored for biosensing to understand different structural and functional changes. This method uses the principle of how the incident polarization state changes on interaction with a tissue or biological sample. Polarimetry has shown promise in the frontier of noninvasive imaging and treatment monitoring (*20*–*22*). Stokes polarimetry, Mueller polarimetry, purity indices from the Mueller matrix, and optical rotation changes based on scattering were examined to determine the contrast difference between abnormal and normal tissues (*13*, *14*). Further polarimetry was used to detect changes in glucose concentration (*18*) and for studying structural changes in skin tissues (*20*). The optical techniques based on polarization for chiral molecular sensing are limited by the penetration depth since most of the studies had used visible wavelength light operating at 589 and 632 nm (*23*, *24*), which is dominated by light scattering effects. CD-based techniques were used to study protein structures and nucleic acids. CD method was efficient in ultraviolet-visible range because the molecular electronic transitions were related to the absorption of circularly polarized light (*25*, *26*). Chiroptical spectroscopy methods were explored for wavelengths up to 1500 nm for photonic nanostructures (*27*). Recently advanced methods for chiral sensing include surface-enhanced Raman scattering (SERS) (*28*) and Raman optical activity (*29*, *30*). Raman scattering–based methods were explored in the visible and infrared (IR; up to 800 cm^−1^) range for applications related to drug testing (*31*, *32*). Since, Raman methods were known to suffer from low–signal-to-noise ratio, SERS tagged the active molecules with metallic nanoparticles to increase the detected signal strength. All chiroptical experimental configurations used revealed information only from the superficial layers due to dominant scattering. Therefore, extracting information about the optical activity with respect to depth became quite challenging with polarization methods.

Photoacoustic (PA) sensing uses the principle of PA effect (*33*) and has been used for multimodal cancer imaging and theranostic applications (*34*, *35*). Earlier works have demonstrated the advantages (like deeper penetration, less scattering, and autofluorescence effects) of developing optical/PA systems while operating at the near-IR II (NIR-II; 1000 to 2000 nm) and mid-IR window compared to visible/NIR region (*36*–*39*). The attenuation in the tissue can be lesser at the NIR-II range as the photon scattering is lesser compared to visible wavelengths (*40*). The attenuation is reduced by 60 to 80% at the NIR-II wavelengths as opposed to the visible wavelengths. These factors indicate a possibility of deep tissue sensing while using NIR-II light (over visible wavelengths). NIR-II PA spectroscopy was devised to estimate biomolecular concentrations using the acquired PA spectrum (*41*, *42*). Polarization properties like dichroism and Mueller matrix were investigated with PA microscopy and PA computed tomography (PACT) (*43*, *44*) while operating in the visible NIR region. These studies enabled better contrast by using the concepts of linear dichroism and anisotropy. Studies with PACT have reported the variations in the PA signal amplitude as a result of varying the polarizer angle (*45*). While another study has shown how linear dichroism changes can be imaged deep inside scattering media by calculating the orientation of the axis from reconstructed image (*43*). The orientation of optic axis from reconstructed images by considering the absorption to be vector in the visible range. Another study (*46*) integrated the concept of the Mueller matrix from the perspective of PA and demonstrated a better signal-to-noise ratio. Using these techniques in the IR range for sensing chiral biomolecules like glucose is less explored. In this work, we have for the first time integrated Malu’s law with PA recording to estimate chiral molecular concentrations, which has a lot of potential for deep tissue sensing. Notably, the existing PA sensing/imaging methods do not consider optical rotation for concentration detection or sensing. Perceiving the challenges in chiroptical sensing and leveraging the advantages of PA, we have hypothesized that chirality-enhanced PA measurements could improve chiral biomolecular sensing, with potential in vivo application.

In this work, we propose a method to estimate the optical rotation of chiral biomolecules in the context of PA, termed as PA-based polarization-enhanced optical rotation sensing (PAPEORS). We analyzed the PA time series data obtained in a transmission mode configuration for sensing concentrations of optically active substances like glucose and naproxen [nonsteroidal anti-inflammatory drugs (NSAIDs) used for relieving pain and muscle aches]. When polarized light interacts with tissue or particle suspensions, the polarization direction rotates, altering the polarization state of the transmitted light. This is primarily due to scatterers in the solution, though optically active molecules also rotate the light and change its orientation. We measure the optical rotation from the PA signal, which is influenced by light fluence and absorption by chromophores as compared to optical detection techniques where the output state of polarization is detected and then further used for sensing and imaging.

The depolarization properties of a biological tissue depend on thickness and other factors, like absorption, refractive index, and the fluence of the incident beam. It is difficult to measure the changes in the polarization at a particular depth using optical detector, as the measured optical signals are often influenced by scattered photons. Therefore, using an ultrasound detector makes it easier to sense from deeper tissues due to the time-resolved nature of the measured acoustic signals.

This study investigated the potential of PAPEORS for the detection of chiral molecules in deep tissue and to address challenges faced during in vivo chiral molecular sensing. To the best of our knowledge, the investigation of optical rotation changes by detecting the PA signals was not explored. PAPEORS was used to: (i) estimate the optical rotation from PA time series measurement using a trans-illumination configuration; (ii) validate the proposed system for accurately sensing chiral biomolecular concentration using optical rotation at larger depths, i.e., about 3.5 mm; (iii) evaluate the optical rotation changes with respect to different polarized incidences: vertical (V), 45° linear (P), and circular (R) polarization; and (iv) conceptualize a noninvasive polarized PA sensing method. This work evaluated the variation in the estimated optical rotation (from PAPEORS) as a function of chiral molecular concentration in aqueous-based glucose samples, serum [bovine serum albumin (BSA)]–based glucose samples, and naproxen. Last, we discussed the prospects of PAPEORS by highlighting a single wavelength usage which can lead to easy miniaturization of the system.

## RESULTS

### Glucose rotation estimation using PAPEORS

**Fig. 1.**
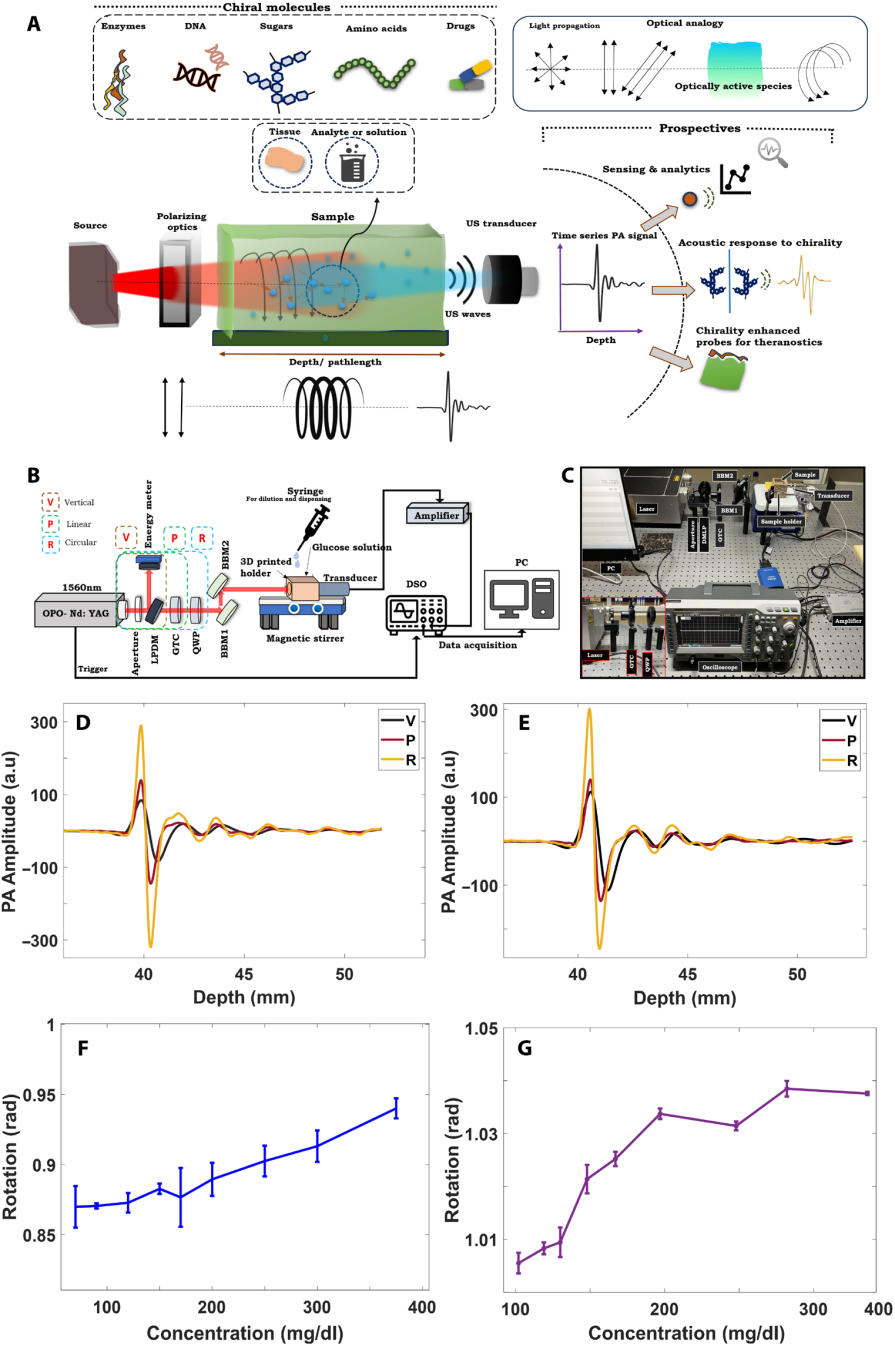
Principle of PAPEORS. (**A**) Schematic describing the concept and potential applications of PAPEORS. (**B**) Schematic of the experimental setup used for optical rotation measurements. The components used for the optical alignment and polarization generation are an aperture of diameter 6 mm to reduce beam diameter. DMLP, long pass dichroic mirror; GTC, Glan-Taylor crystal polarizer; QWP, quarter-wave plate; and two BBM1 and BBM2: broadband mirrors. Energy meter was aligned with DMLP for real-time energy measurements. (**C**) shows the photograph of the experimental setup used for acquiring the PA measurements. (**D**) and (**E**) show the acquired PA signals for the aqueous and BSA glucose solutions, respectively, for V, P, and R incidences for 350 mg/dl concentration. The R incidence shows the best SNR for both samples compared to V and P incidence. (**F**) and (**G**) show the optical rotation analyzed at a depth of 2 mm from the PA experiments for V incidence. (F) shows the optical rotation as a function of concentration for aqueous glucose samples and (G) for the serum-based samples.

The schematic in Fig. 1A highlights the concept of PAPEORS for a wide range of chiral molecules present in human body. The optical analogy to the proposed concept is emphasized on how optical rotation can be visualized from acoustics point of view. The prospectives are listed on the side shows opportunities where PAPEORS can be used potentially. PA signals with three different polarization incidences for optical rotation estimation were obtained using the setup shown in Fig. 1 (B and C), which shows the photograph for the same. The raw PA signals (acquired at 1560 nm) for aqueous glucose sample and BSA-based sample having a concentration of 350 mg/dl for different polarized incidences are indicated in Fig. 1 (D and E, respectively). Details of the experimental system is elaborated in the “PAPEORS experimental setup” section.

After energy normalization, the signal amplitudes for circular and linear polarization states had a higher signal-to-noise ratio than those for vertical incidence. The recorded PA signals were affected by the light attenuation and the transducer response. The deconvolved time series PA signal (as explained in fig. S1 and text S2) was used to estimate the optical rotation.

### PA spectrum for V, P, and R polarized incidence

**Fig. 2.**
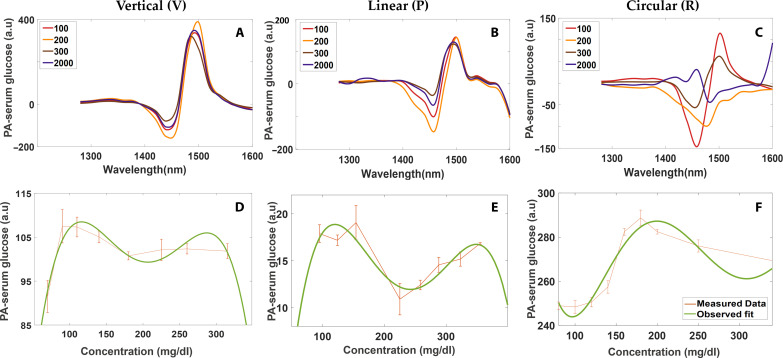
PA spectrum and nonlinear effects with serum-based samples. (**A**) shows the PA spectra for the vertical (V) incidence, (**B**) PA spectrum for Linear (P) incidence, and (**C**) PA spectrum circular (R) incidence. (**D**), (**E**), and (**F**) show the nonlinear variation of the PA amplitude as a function of concentration at a depth of 1.7 mm for V, P, and R incidences, respectively. Nonlinearities were observed from spectroscopic techniques after 1.7 mm depth for the serum-based samples, hence extracting concentration from deeper regions will be difficult.

Three concentrations within the physiological range (100, 200, and 300 mg/dl) and one highly saturated concentration (2000 mg/dl) were chosen for analyzing the PA spectrum with different polarization incidences. The spectra shown in Fig. 2 (A to C) were for serum (BSA)–based samples for V, P, and R incidences. PA measurements were also performed for the aqueous glucose solutions and the spectra acquired is shown in fig. S2 (A to C). The baseline spectrum of water and BSA were recorded separately to enable baseline correction. The glucose peak absorbance was observed from 1500 nm for vertical, linear and circular incidences.

Figure 2 (A to C) clearly illustrates that the PA signal does not show a linear correspondence with an increase in glucose concentration, i.e., the recorded PA signal for the highly saturated concentration (2000 mg/dl) was found to have lesser intensity than 300 mg/dl in all the instances and with multiple repetitions.

PA amplitude as a function of concentration at 1.7 mm depth (from the point of illumination) is represented in Fig. 2 (D to F). Because of light attenuation effects, the recorded PA signals do not linearly increase with concentration; note that such nonlinear behavior was found at depths larger than 1 mm. Figure 2 clearly demonstrates that the linearity fails from spectroscopic techniques after 1.7 mm depth for the serum-based samples. Figure 2 (D to F) shows the nonlinearity for depths greater than 1.7 mm with linear and circular incidence. Similar variations for the aqueous glucose samples are reported in fig. S2 (D to F). At superficial depths, PA spectroscopy will be linear, as the measured PA signal will directly correlate with the moiety concentration. However, the PA signals from larger depths will be heavily influenced by fluence effects, i.e., lower concentration will lead to higher fluence, whereas high concentration will lead to lower fluence at larger penetration depths; this phenomenon will lead to nonlinearities at larger depths while using PA spectroscopy. Hence, we concluded that PA spectroscopy might fail at larger depths, making it difficult to extract the chiral molecular concentration reliably from the recorded PA data. Consequently, we proposed the utility of PAPEORS system to enable more efficient sensing from deeper tissues using single-wavelength measurements.

### Optical rotation of D-glucose from PA measurements

D(+) glucose samples (prepared in aqueous and BSA solution) are dextrorotatory in nature. The recorded PA signal was initially preprocessed and then deconvolved with the transducer response to give a final output signal, which clearly shows the light attenuation signal (fig. S1D). The PA amplitudes were then chosen as described in the “PAPEORS principle” section in Methods and fig. S1. These amplitudes, i.e., P and $P_{0}$, were then used to compute the optical rotation. Figure S1 describes the steps involved in deconvolution of the time series PA signal. The time domain and frequency domain responses of the acquired PA signal and the deconvolved PA signal is represented (fig. S1). The transducer impulse response that has been used for deconvolving the signal to get the fluence profile is shown in fig. S1C.

Optical rotation as a function of concentration at a depth of 2 mm for the V incidence is illustrated in Fig. 1 (F and G). Figure S3 shows the calculated rotation varying as a function of concentration for P and R incidences for aqueous solution (fig. S3, A and B) and serum-based samples (fig. S3, C and D). The changes in rotation observed are in the order of millidegrees to degrees, which aligns well with polarimetric methods (*24*). The trends observed from these measurements were the basis upon which the concentration prediction model was proposed. A linear relationship between rotation and concentration was observed, which corresponds well with optics literature (*47*).

Factors affecting rotation include: (i) water absorption interfering with PA signal, (ii) pathlength choice, (iii) laser energy fluctuations, (iv) solvent-based Brownian motion, and (v) experimental geometry. Owing to these factors, data were collected over multiple days to ensure repeatability (fig. S6, A and B). Figure S6 (A and B) shows that the deviations in rotation measurements over a couple of days for 120, 250, and 400 mg/dl were evaluated. The plots shows deviations highlighted at two different depths, i.e., 2 and 3.5 mm.

Further, polarized Monte Carlo (*48*) simulations were performed to validate the obtained experimental optical rotation estimates from different polarized incidences. We observed a good correlation between the simulated fluence profiles and experimental data obtained at a depth of 1.7 mm with different polarization incidences (fig. S4). The plots in fig. S4 (A to C) highlight the correlation of the magnitude of rotation between the experiments and simulations for V, P, and R incidences for a depth of 1.7 mm. The absorption coefficient was varied on the basis of the molar absorptivity of glucose at 1560 nm for including the optical rotation, and the scattering was set to be low considering that scattering is less at this wavelength.

Further experiments were performed to study the interference of other molecules. Water has an absorption peak at 1440 nm and albumin is known to have a peak close to 1490 nm (*49*). Glucose is known to absorb much stronger from 1560 nm than water. Hence, 1560 nm was chosen for our preliminary experiments. We also calculated the optical rotation experimentally from the recorded PA data for water and BSA samples. Figure S7 shows that the rotation of glucose is dominant at 1560 nm compared to stand-alone BSA and water sample. Hence, this system could enable accurate in vivo sensing, as this work considers optical rotation at 1560 nm to detect concentration compared to other methods. Further, analysis was also performed to assess how the rotation angle changes with an increase in path length. The plot is summarized in fig. S8 for a depth of range of 0 to 5 mm.

In tissue polarimetry, a minimum of four to six measurements are required for forming the intensity images representing the Stokes vector. Stokes vector represented by $S={\left[\text{IQUV}\right]}^{T}$ represents the total intensity (*I*), horizontal or vertical component (*Q*), −45° or +45° linear (*U*), and left or right circular (*V*) (*14*) polarization. The Mueller matrix is a 4 × 4 matrix (having 16 elements) that indicate the transformation of the polarization states (*14*). The elements M22, M23, M32, and M33 contain more information about rotation and can be used to determine if the species evaluated is chiral in nature than the other elements (*18*). The corresponding incident illumination for these elements is vertically or linearly polarized incidence. Hence, our initial experiments used vertical and +45° linear polarization to understand the effect of vertical and linear illumination on optically active medium. Circular polarization (right handed or left handed) rotates just the plane of polarization to the phase (from *R* to *L* or *L* to *R*). The interesting part about that is that circular polarization can go deeper and survive more scattering, so it is known to have a “memory” (*13*, *50*). We were keen to study the effect of circular polarized incidence on the optical rotation and how deep it can penetrate in the context of PA. Therefore, these V, P, and R polarized states were chosen for our initial experiments. For completeness, a brief study was conducted to evaluate how the rotation varied for the other polarization incidences: Horizontal (H), −45° linear, and left circular (L) polarization states. Figure S11 shows the rotation calculated as a function of concentration for the H, M, and L polarization states for a path length of 1.7 mm. A total of 10 glucose samples dissolved in BSA were evaluated for each of the incidences and repeated three times to check the variations in rotation. Figure S11 (A to C) shows that the order of magnitude of rotation is slightly different for all the polarized incidences. This can be attributed to the fluence incident and the Brownian motion of the particles. The experimental details are explained in Methods.

### Concentration estimation from the rotation of D-glucose

**Fig. 3.**
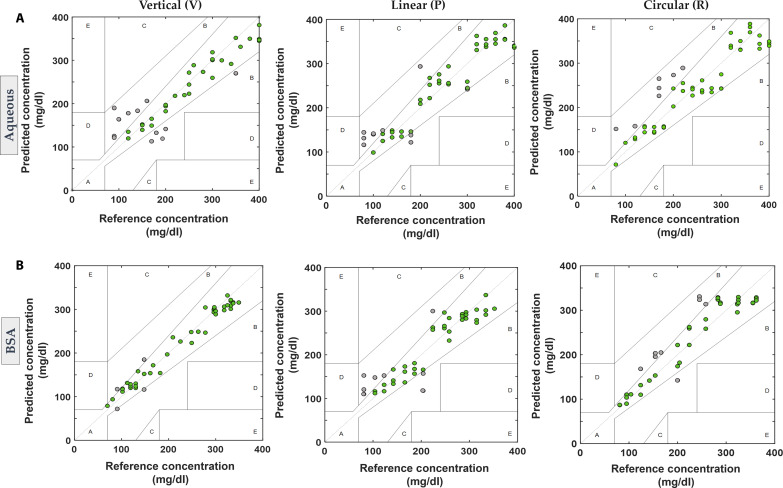
CEGA for Glucose estimation using PAPEORS. (**A**) Top row shows the CEGA for the aqueous glucose samples for V, P, and R polarized incidences respectively. (**B**) Bottom row shows the CEGA for serum-based glucose samples for the V, P, and R polarized incidences, respectively. The BSA glucose samples for the vertical incidences shows the highest percentage of predicted points in zone A. Overall, serum samples show a higher percentage of predicted concentrations in zone A than the aqueous samples for all three polarized incidences.

A quadratic regression model was used as indicated in Eq. 6. Approximately three outliers per dataset were eliminated while building the model during the analysis. Data acquired from the same day was used to build the model and perform the estimation. Figure 3A shows the Clarke’s Error Grid Analysis (CEGA) for the aqueous and Figure 3B shows the CEGA for serum-based glucose samples with V, P, and R incidences. The concentration estimation (with serum-based samples) from the vertical incidence had about 87% points in the zone A, while linear incidence had about 79% points. The circular incidence had 82% of the total points lying in zone A of CEG (Fig. 3B). The important parameters derived are summarized in Table 1. Few estimated points for the aqueous samples case fell in zone B, because the estimated rotation had high variance as a result of dominant water absorption. The accuracy of the model could be further improved using advanced machine learning models.

**Table 1.** Estimated parameters for ex vivo experiments and CEGA of glucose solutions. The percentage of predicted concentrations falling in the zone A of the CEGA and the estimated limit of detection (LOD) is shown for the aqueous glucose, serum glucose samples, and ex vivo chicken tissues. The parameters are summarized for V, P, and R incident configurations. The best LOD was observed for circular incidence for the solution samples. The best predicted concentrations are seen for the vertical incidence data and the lowest LOD for circular incidence at a depth of 2 mm for the ex vivo experiments.

Incident polarization	Vertical		Linear		Circular	
Solution	Aqueous	BSA	Aqueous	BSA	Aqueous	BSA
Zone A %	62.5%	87.5%	87%	79.3%	82%	84.4%
LOD	120 mg/dl	107 mg/dl	98 mg/dl	104 mg/dl	80 mg/dl	81 mg/dl
Ex vivo	BSA		BSA		BSA	
2 mm zone A %	90.9%		86.7%		81.5%	
LOD	91mg/dl		90mg/dl		85mg/dl	
3.5 mm zone A %	83%		84%		83%	
LOD	91 mg/dl		90 mg/dl		84 mg/dl	

The limit of detection (LOD) for the predicted glucose levels was determined for each polarized incidence based on points falling in zone A of CEG. Notably, the detection limit for V incidence data is the worst at 120/107 mg/dl compared to P and R incidences for aqueous/BSA samples. The circular incidence data yielded the lowest predicted limits for aqueous and BSA samples at 80 and 81 mg/dl.

### Ex vivo PAPEORS experiments for deep-tissue glucose detection

**Fig. 4.**
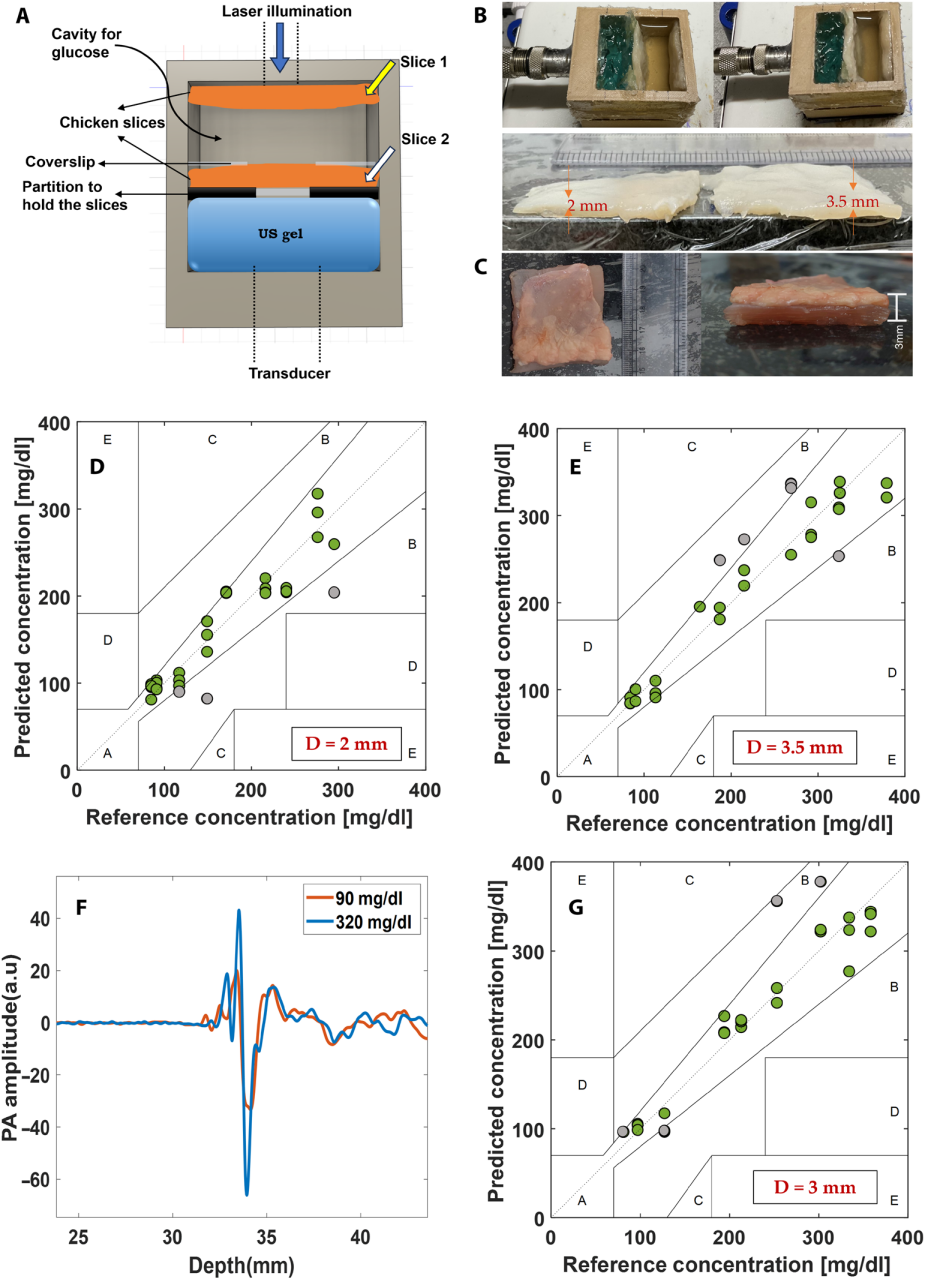
Ex vivo studies for serum-based glucose concentration estimation. (**A**) shows the schematic of the holder and the positioning of two chicken breast slices during PA measurements at two different depths: 2 and 3.5 mm; (**B**) shows the dissected slices used for the experiments and placements of the same during measurements; (**C**) shows the chicken slice with a layer of skin; (**D**) and (**E**) show the CEG for the vertically polarized incidence for 2 and 3.5 mm, respectively; (**F**) shows the PA signal acquired for a low and high concentration from the sample with skin having thickness 3 mm; and (**G**) indicates the CEG for the sample with skin.

Ex vivo PA experiments were performed using thin slices of chicken breast. The details of the sample preparation are elaborated in the “Glucose sample preparation” section. The configuration used for data acquisition can be seen in Fig. 4 (A and B).

The tissue slices were placed perpendicular to the axis of illumination and were held by a fixture at the bottom of the holder. The space between the two slices was filled with a serum-based glucose solution having different concentrations (Fig. 4B).

This kind of configuration was attempted to mimic an in vivo scenario from where we can detect the blood glucose levels inside thick biological tissue samples noninvasively. The ex vivo sample was designed to mimic a real scenario, enabling noninvasive detection of glucose from deep tissues. Figure 4A illustrates the configuration of the entire ex vivo setup used in the experiments. The first slice, placed parallel to the laser illumination, helps evaluate the penetration of the laser into the tissue and aids in detecting glucose concentration from a depth. The slices were held upright (see Fig. 4B) in the holder to maintain proper orientation. The thickness of the slice placed close to the illumination source (Fig. 4A) was varied, i.e., 2 and 3.5 mm, to assess the system’s penetration and its ability to generate acoustic signals. The slices of thickness 2 and 3.5 mm dissected for the experiment can be seen in Fig. 4C. PA signals for 10 glucose concentrations from 90 to 400 mg/dl were recorded at 2 and 3.5 mm depths. First, the rotation was calculated, and then the concentrations were predicted using the quadratic fitting model (Eq. 6) for V, P, and R incidence configurations. The predicted glucose concentrations from the ex vivo experiments can be seen in Fig. 4 (D and E) for the V incidence at 2 and 3.5 mm, respectively. 90.9% of predicted concentrations fell in zone A of the CEG for the vertical incidence for 2 mm and 83% for 3.5 mm. CEGA for the P and R incidence configurations is shown in fig. S9. Table 1 summarizes the parameters observed from the CEGA for ex vivo experiments with all three polarized incidences using PAPEORS.

Further assessment of the LOD variation as thickness varies was also studied. Figure S10 shows the comparison of the LOD for different depths for V, P, and R incidences. The data from ex vivo experiments were used to observe how the LOD varied with thickness and for different polarized incidences considered (fig. S10 B). Also, the LOD variations were assessed at four different depths (between 0.26 mm to 3.5 mm) for serum glucose samples to see how LOD varies with depth. For evaluating the effects of scattering from skin, another experiment was performed with a chicken tissues slice with a layer of skin having thickness of 3 mm. The PA signal is noted to have slightly more fluctuations, which could be due to the skin layer. The skin layer used for the experiments was also observed to have a nonuniform distribution of fat. The results are shown for vertical incidence in Fig. 4 (F and G). A total of 85% of the total number of predicted concentrations fall in the zone A of the CEG. Although the accuracy is noted to drop slightly compared to the samples without a layer of skin, there was no hindrance to mapping the fluence from the acquired PA signals and further concentration estimation using optical rotation.

### Optical rotation of naproxen using PAPEORS

**Fig. 5.**
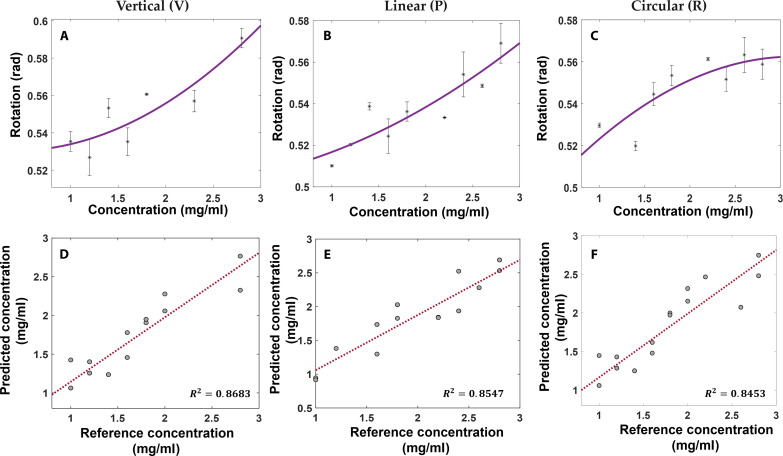
Optical rotation and concentration predication of naproxen using PAPEORS. (**A**), (**B**), and (**C**) showcase the variations in V, P, and R incidences for the Naproxen solution having the concentration range of 0.8 to 2.8 mg/ml. The experiments were performed at 1500 nm as that was identified to be the peak absorption from PA spectrum. The linear increase in the magnitude of rotation with the increase in concentration can be observed. On the basis of this, the linear regression model for concentration prediction was built. (**D**), (**E**), and (**F**) show the predicted concentration using linear regression based on the variations in the rotation. The $R^{2}$ values are indicated as inset in (D) to (F).

Naproxen is an NSAID drug used to treat mild to severe pain and inflammation. This molecule is known to exhibit D+ rotation (*51*). The spectrum was characterized for naproxen at three different incidences to identify the absorption peak. After the blank correction with ethanol, an absorption peak was observed at 1500 nm. The recorded PA spectra for naproxen with V, P, and R incidences are shown in fig. S5. The spectra before the blank (70% ethanol) correction are in fig. S5A, while postcorrection spectra are in fig. S5B, highlighting the naproxen absorption peaks for each incidence. Rotation experiments were performed by diluting a high-concentration naproxen, i.e., 2.8 mg/ml with 70% ethanol. Figure 5 shows the variations in optical rotation observed with changes in naproxen concentration using PAPEORS. Figure 5 (A to C) shows the rotation trends observed with different polarized light incidences. The rotation for naproxen was calculated at a depth of 0.5 mm, beyond which fluctuations started to amplify.

Since the optical rotation was varying linearly with concentration, a single linear equation was used to build the fitting model, unlike the glucose case. The reference concentration was considered on the basis of the prepared weight-to-volume ratio. The predicted concentrations (using the linear fitting model) were plotted against the reference concentrations, and the goodness of fit was evaluated, as shown in Fig. 5 (D to F). The $R^{2}$ values are also shown as the inset of Fig. 5.

### Pilot studies demonstrating in vivo feasibility

**Fig. 6.**
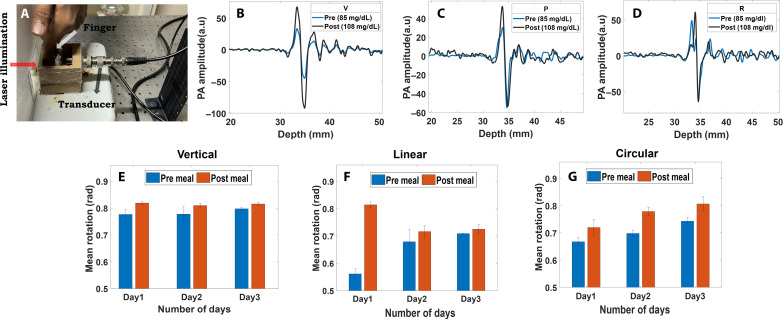
Pilot studies demonstrating in vivo feasibility. (**A**) Noninvasive experimental setup using PAPEORS. The positioning of the finger can be seen here. (**B**), (**C**), and (**D**) show the recorded PA signals acquired premeal and postmeal for V, P, and R incidences. The estimated optical rotation from the time series data can be seen in (**E**), (**F**), and (**G**) for 3 days for V, P and R incidences.

A proof-of-concept noninvasive glucose detection study was performed to evaluate the efficacy of the proposed PAPEORS framework. The experiment was designed to illuminate a partial region of a finger using the same custom-designed holder and the positioning for the noninvasive measurement can be seen in Fig. 6A. The experiments were performed preprandial and postprandial on three different days from the same volunteer. Before conducting the experiments, informed consent was obtained from the volunteer in accordance with the IHEC (Institutional Human Ethics Committee) guidelines. Ultrasound coupling was ensured using deionized water in the holder. The PA signals acquired pre- and postmeal were shown in Fig. 6 (B to D) for V, P, and R incidence, respectively. The recorded PA signals increased in the postmeal case due to increased glucose concentration, which corroborated with the glucometer reading obtained as the reference measurement using finger prick method (as per protocol approved by IISc IHEC/03/05.04.2024). We further evaluated the optical rotation from the recorded PA measurements. The optical rotation was found to increase postprandial owing to an increase in glucose level postmeal. The increase in the estimated optical rotation was consistent with all the incidences and can be noted in Fig. 6 (E and F) for day 1, day 2, and day 3. These observations show that PAPEORS can be effectively extended to build a concentration prediction model that will predict glucose concentration accurately in a noninvasive manner.

## DISCUSSION

PAPEORS has the ability to measure the optical rotation at unprecedented depths in biological tissue/turbid media. Our work revealed that there are variations in the observed PA signals when the incident state of light polarization was changed. CEGA for glucose sensing suggests the potential application of PAPEORS for continuous blood glucose monitoring, which is critical for diabetes management (*52*). PA imaging was indeed used earlier for direct glucose sensing (*42*) and assessing diabetes-related risk factors by studying vascular structures (*53*). Recent advancements in PA include sensing noninvasive glucose using IR PA microscopy, where the concentrations are detected by time gating the signals and mapping them to the depth, which offers insights on how the concentration detection varies at different depths and how influence from spectral components of skin can be considered for detection (*54*). Polarized PA microscopy has been studied for quantifying differential absorption of fibrous tissues (*55*) and visual enhancement of biological structures based on their anisotropy and orientation (*56*). PA measurements were used in the context of Stokes and Mueller matrix–based analysis to image amyloid plaques in an Alzheimer’s brain (*57*). PAPEORS can be customized to provide direct in vivo glucose information making it a strong candidate to solve the holy grail in diabetes management.

PAPEORS can be developed as a straightforward, noninvasive system, which will not require complex sample preparation steps like the conventional chiral sensing methods (*10*). Chiral molecular sensing using PAPEORS was found to be more accurate than PA spectroscopy (due to nonlinearities) at larger depths. The time series PA measurements allow us to precisely sense from a specific region/depth of the tissue. PAPEROS sensed glucose at a depth of 2 mm (with chicken breast slice) having LOD as 85 mg/dl using circularly polarized light incidence. Table 1 shows the estimated parameters for ex vivo experiments, which indicates PAPEORS’ effectiveness even with complex biological structures and potentially be useful for developing a noninvasive continuous glucose monitoring (CGM). Noninvasive CGM is very crucial for diabetic patients to understand/regulate the treatment plan and management to prevent complications. Having a real-time CGM system or device is very relevant for patients in critical care units (*58*). Studies have also shown how CGM plays a role in obesity research and management (*59*). This work focuses on clinically significant concentration range for CGM is 70 to 400 mg/dl (*60*). We were able to estimate concentration levels from 80 mg/dl with our proposed quadratic regression model without any complex algorithms. In essence, circular polarization could be used when evaluating lower (hypoglycemic ranges) concentrations, as we can get minimum LOD from the experiments. The vertical and linear polarization incidence can be preferred for hyperglycemic ranges as we would be able to evaluate the optical rotation changes more distinctly. Detecting concentrations lower than 80 mg/dl has its own challenges. Notably, the patients’ pool with hypoglycemic ranges is also limited, as lower concentrations of glucose tend to become symptomatic, having fatigue, light-headedness, and excess sweating. So, the aim of this sensor is to identify abnormal glucose levels noninvasively and provide appropriate intervention in terms of insulin dosage estimation. Further advanced machine learning methods can be integrated as part of PAPEORS to improve sensitivity and prediction accuracy. Also, system optimization in terms of light delivery and multiple acoustic sensors could allow for improving LOD.

We have overcome the scattering effects by using NIR-II wavelengths, i.e., 1560 and 1500 nm that allows deeper penetration. We have also shown that the interference from water and BSA are negligible compared to the magnitude of optical rotation by glucose. We have used serum based glucose samples which contain albumin, which had not influenced the estimated optical rotation. The scatterer size has been characterized for different types of albumin in biological tissues (*61*).

Moreover, an optically active species has a structural geometry that enables it to rotate the plane of polarized light. For instance, it is well known that a linearly polarized incidence will become circularly polarized on interacting with the optically active species irrespective of other scatterers. The scattering that occurs in a turbid medium can be as result of mild refractions in tissue. As these are originated from individual molecular scatterers, the polarization properties might change. However, if the interaction is with an optically active medium, the entire plane of polarization is rotated whether there is turbidity or not in the biological samples (*47*). The presence of other scatterers might affect the intensity of transmission and depolarization; however, the scattering is very less in NIR-II wavelengths, minimizing the effect of other scatterers. Thus, using NIR-II wavelengths for measuring optical activity of glucose concentrations in plasma solutions containing BSA and glucose can yield accurate results, closely resembling blood plasma conditions. Currently, PAPEORS uses a single wavelength for concentration estimation, which is highly advantageous in miniaturizing the system for a focused sensing application. The biological fluids contain more than one functional molecule of interest. Therefore, signal interference from these molecules like water, lipids, and proteins might affect the measurements. Hence, it is recommended to perform initial spectroscopy, which can reveal the wavelength having higher absorption corresponding to specific moiety of interest. We have indeed demonstrated realistic ex vivo experiments with serum-based samples, indicating PAPEORS’ potential to sense chiral molecules with limited interference. Experiments were repeated multiple times to ensure repeatability.

PAPEORS analyzed the optical rotation from PA signals and demonstrated a good sensing method; however, it was not devoid of limitations. First, (i) serum-based glucose sample was investigated as they closely mimic the blood plasma. Further, ex vivo configuration was attempted to replicate a controlled environment compared to in vivo setting (as glucose concentration is known to vary drastically in vivo). These experiments gave foundational insights into the optical rotation estimates from PAPEORS. We have performed preliminary noninvasive measurements and concluded that optical rotation increases when the glucose concentration increases. However, detailed in vivo studies with PAPEORS should be performed to evaluate its reliability; specifically, a regression model needs to be developed and validated for the in vivo measurements in terms of concentration estimation, which will be taken up as future work. Second, (ii) multiple chiral molecules are present in the human body and are significant biomarkers. Characterizing them and sensing them simultaneously were not explored using PAPEORS. PAPEORS was indeed used to demonstrate sensing of multiple chiral molecules by using different wavelengths (corresponding to the absorption peak of each moiety). Hence, it is a straightforward extension of PAPEORS to study mixtures of chiral molecules.

The speed of sound (SOS) variation for the different layers in the ex vivo tissue samples was currently not considered, and we assumed the homogeneous SOS for all the layers for simplicity. SOS can be calibrated postdata acquisition using methods that use autofocusing algorithms and focus metrics on determining the best SOS without having mismatch between the original SOS of the tissue (*62*). There are deep learning–based SOS optimizations that can mitigate the discrepancy/noise caused by SOS variations. This was accomplished using deep neural networks, specifically through the DeepMB model, which enabled dynamic variations in the SOS in the model and enhanced the quality of the resulting PA data (*63*). Techniques that include ultrasound information as “soft priors” in the PA reconstruction methods can help compensate for the changes in SOS variations for different tissue types and improve the PA data, especially when the SOS of the medium/tissue is not well known (*64*).

PAPEORS sensing can be extended to investigate and quantify the specific rotation of chiral particles, CD, and birefringence at a deep tissue level and explore the impact of incident polarized light rotation on the generated acoustic signals. Currently, PAPEORS was evaluated at NIR-II wavelengths; however, similar concepts can be explored at other wavelengths like mid-IR, terahertz, etc. PAPEORS has potential for compact wearable integration (*65*) and coupling with endoscopic probes for high resolution PA imaging enabling gastrointestinal cancer/lesion detection (*66*). Since, PAPEORS offer deeper penetration of light and good contrast, it holds promise in the context of multimodal imaging and combined therapies.

## METHODS

### PAPEORS principle

The optical activity of the chiral molecule is measured in terms of the rotation of the molecule. The optical rotation of the molecule depends on the incident state of polarization, concentration (*c*), path length (l), and the wavelength (λ) of light. The relation between concentration and optical rotation (θ) is given as (*67*)

$$c=\frac{\theta }{{{\left[\theta \right]}_{\lambda }^{T}}^{*}l}$$ (1)

where ${\left[\theta \right]}_{\lambda }^{T}$ is the specific rotation of the chiral molecule and is dependent on wavelength and temperature. The pressure generated by a chromophore as a result of the absorption at a certain wavelength using the PA effect is given as (*41*)

$$P={\tau \mu }_{a}\phi$$ (2)

The generated PA signal is proportional to the Grüneisen parameter (τ), absorption coefficient ($\mu_{a}$), and light fluence (ϕ). We propose a method to combine and study the optical rotation of glucose/naproxen by inferring the fluence from the recorded PA time series signals. For combining the optical and PA phenomena, we have considered the Malus’ law, given as

$$I=I_{0}{\text{cos}}^{2}\theta$$ (3)

In general, I is considered as the intensity transmitted and $I_{0}$ is the incident light intensity. This work has correlated the light fluence at different depths to the time series PA signal, such that it is analogous to the Malus’ law representation, as follows

$$P=P_{0}{\text{cos}}^{2}\theta$$ (4)

where P and $P_{0}$ denote the amplitudes of PA signal and are chosen on the basis of the depth or the path length of the light travelled. Last, the rotation (θ) experienced by the chiral molecules can be computed from the above equation once P and $P_{0}$ are known using

$$\theta ={\text{cos}}^{-1}\sqrt{\frac{P}{P_{0}}}$$ (5)

The recorded PA signal can be directly correlated to the light fluence (with an assumption of having a constant Grüneisen coefficient) due to lesser scattering effects at the NIR-II window.

$P_{0}$ was chosen close to the illumination point on the holder (approximately 0.018 mm), and P was chosen at a particular depth depending on the path length of interest (1.5, 2, or 3 mm). The detailed steps involved in extracting the points from deconvolution are described in fig. S1. On the basis of the optical theory, rotation is observed to increase linearly with the concentration (*14*, *47*). However, we observed a slightly nonlinear behavior for the rotation at lower concentrations (i.e., from 70 to 160 mg/dl) and good linearity for higher concentrations (refer to Fig. 1, F and G). Assuming linear variations led to inaccuracies in the predicted concentrations from our experiments. The trends observed in the rotation, which was mostly quadratic in nature, influenced the concentration prediction model. Hence, a quadratic regression model was used for concentration estimation. The details of the model used for the concentration prediction ($C_{p}$) obtained from the observed rotation (θ) is explained in the “Noninvasive PAPEORS experimental setup” section.

### PAPEORS experimental setup

The optical rotation of the glucose samples was studied using the setup shown in Fig. 1B. The glucose solution placed in a custom-designed three-dimensionally (3D) printed holder was illuminated using a nanosecond pulsed Nd:yttrium-aluminum-garnet laser (Innolas SpitLight OPO; wavelength of 1100 to 1700 nm, 30 Hz, and 7 ns). The PA signals were recorded using a single-element ultrasound transducer having a central frequency of 7.5 MHz (Focused, Olympus V320-SU-F1.2IN-PTF, 60% bandwidth) fixed in the 3D printed holder containing the sample. The focus of the transducer used is 1.2 inches. The schematic in Fig. 1B shows the configuration used for acquiring the optical rotation measurements. The rotation experiments were performed in a trans-illumination configuration. An aperture of 6 mm diameter was positioned at the laser output to reduce the beam diameter. The beam diameter was reduced to be within the damage threshold values of the polarization optics used in the experiments for alignment. A long-pass dichroic mirror from Thorlabs (DMLP650) was used in the beam splitting configuration. An external energy meter (Newport Pyroelectric energy sensor) was used to record the energy falling on the sample for each incident state of polarization. The energy meter was kept in the path of the reflected ray from the DMLP and the transmitted ray through DMLP was allowed to pass through the rest of the optics and then to the sample. The acquired time series PA signals were normalized with the energy measured in real time using an external energy meter. A total of 60 (30 on the same day and 30 on the second day) readings were acquired on the same day and multiple days to ensure the reliability of the setup designed for V, P, and R incidences (fig. S6, A and B).

The laser beam was further aligned using two broadband mirrors (BBMs; Thorlabs, BB1-E04) inclined at 45° to each other to adjust the working height for the data acquisition configuration. Different states of polarization was considered as input, and initially, a vertically polarized light was directly used as an illumination source for PA measurement acquisition. In the second configuration, the vertically polarized light was used to generate a linearly polarized light, and this was achieved by placing a Glan-Taylor crystal (GTC) polarizer (Thorlabs GT-10C), for introducing a phase shift to the originally vertically polarized light of the laser to become linearly polarized illumination source. The GTC was rotated by an angle of 45° with respect to the axis. Final configuration involved generation of circular polarized light (as illumination source) by placing a quarter-wave plate (QWP) (Thorlabs AQWP05M-1600) in front of the GTC. The transducer was positioned at a depth of 4.1 cm from the point of illumination fixed with silica gel to prevent leakage of the glucose solution. Further to the V, P, and R polarization states, Horizontal (H), −45° linear (M), and left circular (L) states were studied. Experiments were performed by rotating the transmission axis of the crystal polarizer (GTC) and QWP orientations for −45° linearly polarized and left circular states. The laser output at 1560 nm is vertically polarized. The setup in Fig. 1 was modified for the H state by extracting the horizontal component of the transmitted circularly polarized light by using another crystal polarizer (Thorlabs, GL10). The illumination side of the holder was covered with a coverslip and silica gel.

The time series PA signals were collected using a preamplifier (Olympus, PR-06-04) having a 40-dB gain through an oscilloscope (RIGOL MSO2102A) at the rate 1-giga samples per second. Postrecording, the signals were processed by filtering and a series of operations, which are described in the next section. The energy per pulse at 1560 nm from the laser was 2.5, 1.18, and 1 mJ for V, P, and R, respectively. The peak energy densities from the laser were calculated to be 0.04, 0.02, and 0.015 J/cm^2^ for V, P, and R incidence, respectively, which is within the American National Standards Institute (ANSI) limits and did not cause any damage to the tissue used for the ex vivo studies.

The impulse response function of the transducer (Olympus V320-SU-F1.2IN-PTF; focus, 1.2 in) was measured using a different experiment with a water tank and an additional transducer having the same frequency was used to generate an ultrasound pulse. The generated ultrasound pulse was detected by the transducer (Olympus V320-SU-F1.2IN-PTF; focus, 1.2 in), which is used to estimate the impulse response of the transducer. The amplifier used for pulsing and receiving the pulses was the same amplifier used for PA data acquisition (Olympus, PR-06-04). Figure S12 shows the impulse response obtained experimentally and the manufacturer-provided response based on the described setup.

### Noninvasive PAPEORS experimental setup

Noninvasive experiments were carried out by modifying the experimental setup in Fig. 1 (B and C) by introducing a second aperture after the polarization optics to further reduce energy and beam diameter to be within the ANSI safety limits. The energy for each incidence was measured after the second aperture, and maximum permissible exposure was calculated for skin exposure. A finger was positioned in the custom holder as shown in Fig. 6A. The exposure and acquisition time was limited to 15 to 20 s. A different finger was irradiated for the preprandial and postprandial experiments. The energy per pulse was noted to be 85, 150, and 200 μJ, for circular, linear, and vertically polarized light illuminating the finger. The energy density irradiated was calculated to be 1.6, 2.08, and 3.5 m*J*/cm^2^ for circular, linear, and vertical incidences, respectively, which is much lesser than the ANSI safety limit for 1560 nm (*68*). The energy was recorded during each measurement and used for normalization postfiltering. Care was taken that no stray scattering or extra exposure to other regions was there. Note that the transducer was aligned to have focus within the finger region to improve the SNR of the recorded PA signal. The experiments were conducted for three different days for V, P, and R incidences. Three measurements were recorded for each incidence for statistical analysis. The postprocessing part remains the same for the estimation of optical rotation from the time series data. After the experiments, a reference glucose measurement was performed using a finger prick using the glucometer (as approved by IHEC protocol number 03/05.04.2024).

### Postprocessing and concentration estimation

The PA signals recorded during experiments were processed over a series of operations for both spectra and optical rotation estimation. For the spectral data case, the PA signals were filtered using a Chebyshev filter with bandpass limits of 1 to 9 MHz. Peak-to-peak amplitude of the PA signal was considered across the wavelength range of 1280 to 1600 nm. Baseline spectra of water and BSA were subtracted for each sample. The energy recorded at different wavelength during the experiments was used to normalize the PA spectrum data.

The time series data collected for the rotation measurements were also filtered using the same Chebyshev filtering parameters. The data are recorded using a digital oscilloscope at a sampling rate of 1-giga samples per second. The signal recorded contained 1400 time samples. The time samples were converted to time by calculating the appropriate sampling frequency. The time axis of the signal was converted to depth by using the SOS relation to depth and time. The SOS is crucial in establishing the correlation between time and depth. A brief assessment of SOS was conducted to determine and correlate the actual physical depth of the custom holder. The adjusted SOS values were 1420 m/s for the solution-based glucose samples and 1490 m/s for the ex vivo chicken tissue experiments. The adjusted SOS values are obtained by correlating it to the time point of the PA signal generated and the known physical depth from the transducer in the custom holder. If the SOS was not adjusted, we observed inaccurate mapping of the fluence with depth, which will lead to inaccurate calculation of the angle of rotation. Initially, the SOS was assumed to be 1500 m/s before the corrected values were calculated. The discrepancy is due to variations in medium properties. SOS was varied from 1400 to 1550 m/s. The SOS matching the actual physical depth of the custom holder (i.e., 40 mm from the illumination point to the transducer) was chosen for further analysis and postprocessing. Some methods can be applied to the data for estimating optimal SOS using coherent factors from PA images (*69*) or conventional ultrasound (US) algorithms like k-wave and methods integrated with deep learning (*70*). The filtered signals were observed to be convolved outputs of the PA signal with the transducer response. A deconvolution method was opted to correct for the transducer response from the recorded experimental PA measurements. The steps involved in the process of deconvolution are shown in fig. S1. The quadratic regression model for predicted concentrations $[C_{p}\left(\theta )\right]$ based on the rotation versus concentration profile was

$$C_{p}\left(\theta \right)=\left\{\begin{array}{cc} a_{1}\theta^{2}+b_{1}\theta +k_{1}, & \theta \leq \theta_{T}\\ a_{2}\theta^{2}+b_{2}\theta +k_{2}, & \theta >\theta_{T} \end{array}\right.$$ (6)

where $\theta_{T}$ is the threshold value for rotation. $a_{1}$, $b_{1}$, and $k_{1}$ and $a_{2}$, $b_{2}$, and $k_{2}$ are the coefficients of the quadratic equations being used for the concentration prediction. The values were decided on the basis of the variations observed in the rotation detected from the PA measurements for a given path length and the range of concentrations. The value of $\theta_{T}$ was chosen close to 200 mg/dl with rotation magnitudes of 0.88, 0.98, and 0.822 rad for serum samples, and 0.88, 1.025 and 0.97 rad for aqueous glucose samples. The predicted concentrations ($C_{p}$) were obtained from the observed rotation (θ). All the signals acquired were normalized with the energy values measured using the energy meter. All the scripts for analysis and postprocessing were written using MATLAB R2020b. Note that a linear model was used for estimating the concentration in the case of naproxen.

### Glucose sample preparation

Weight-to-volume metric calculations were used to estimate the weight of D+ glucose (SIGMA-CAS 50-99-7) required to be added. The aqueous glucose solutions were prepared by dissolving the weighted glucose in deionized water using a magnetic stirrer plate. Serum-like samples were prepared by dissolving BSA (Himedia, fraction V BSA) in deionized water and then adding the weighed glucose corresponding to each concentration to mimic the nature of blood plasma. The concentration of BSA prepared was 4 g/dl, which falls under normal human serum albumin levels (normal range, 3.4 to 5.4 g/dl). The glucose concentration range was chosen to be from 50 to 400 mg/dl, which lies within the physiological range and one highly saturated concentration of 2000 mg/dl. A stock of 2000 mg/dl was prepared for the spectrum measurements, and a stock of 400 mg/dl was considered for the optical rotation measurements. PA spectrum was acquired with four different concentrations, i.e., 100, 200, 300, and 2000 mg/dl. The concentration of 400 mg/dl was diluted by adding equal parts of water dispensed such that all the concentrations within the physiological range were covered for optical rotation measurements. The solution volume used in the custom-designed holder was 55 ml. All the samples were prepared at least 2.5 hours before the experiments at room temperature. The BSA-based samples were filtered using a syringe filter with a pore size of 0.22 μm. The samples prepared were refrigerated in 4°C postexperiments. Care was taken to bring up the temperature from 4°C to ambient temperature before the experiments were repeated. For the glucose validation measurements, a commercially available glucometer (Accu Chek Verio) was used. A volume of 20 μl of each sample was pipetted out and placed on a petri dish, which is similar to the volume of blood produced on a finger prick to inject onto the glucometer strip.

### Ex vivo sample preparation

Freshly procured chicken breast tissues were used for the ex vivo experiments. The tissue was sliced into very thin slices using a dissection blade. Slices of thickness 2 and 3.5 mm were dissected, as shown in Fig. 4 (B and C) and were used for the experiments. The slices were placed at positions and were held by a fixture at the bottom of the holder. A solid partition was made out of glue. The fixtures were held in place by silica gel for proper bonding. The space between two slices was filled with glucose solution dissolved in BSA of 4 g/ml. Concentration measurements were carried out here by varying the solution concentration from 400 to 90 mg/dl. The volume of sample used for these experiments was 15 ml. US gel was filled within the space between the chicken slice and the transducer after the alignment was ensured. The experiment using PAPEORS was repeated twice to evaluate the system and collect more data points (60 measurements for V, P, and R together).

Freshly procured chicken tissue with skin was used for the experiments to study the effect of scattering from skin. The tissue was dissected to have 3 mm thickness along with the skin. Experimental setup used was similar to samples without skin was used for acquiring the data.

### Naproxen sample preparation

Pharmaceutical testing grade naproxen from Merck (N8280) was used for the rotation experiments. The drug was dissolved in ethanol as the drug was not soluble in water. Therefore, a 70% (v/v) ethanol was used as the solvent. A stock solution of 3.8 mg/ml was prepared, and lower concentrations were evaluated by diluting the stock with 70% (v/v) ethanol. Consumption of naproxen is prescribed in terms of dosages. The drug distribution in the body using standard dosages is about 0.1 mg/ml. The data acquired consist of three sets for each incidence. A total of 90 PA measurements were performed as part of the rotation experiments. PA spectrum were acquired with naproxen sample to identify the peak absorption wavelength.

### Statistical analysis

Statistical studies were performed using the glucometer readings corresponding to the data from multiple sets of experiments. The estimated concentrations using PAPEORS and the glucometer readings were analyzed using a CEGA approach for the glucose samples with multiple repetitions over large concentration ranges. The CEG was plotted by considering a total of 45 measurements repeated three times for the physiological range of 90 to 400 mg/dl, and the reference readings were obtained from the glucometer (Accu Check Verio) for the serum-based samples. The glucometer measurements for the aqueous samples were omitted as the readings displayed a substantial deviation (fig. S6C) from the expected concentration levels, and the known prepared concentration was used as a reference. The deviations from the prepared concentrations for the aqueous and BSA glucose samples are shown in fig. S6C. The mean and SD were used while computing the optical rotation using PA measurements for both glucose and naproxen samples.

## Supplementary Materials

### This PDF file includes:

Supplementary Text Tables S1 and S2 Figs. S1 to S12 References
