# Supplementary material for: Deep tissue sensing of chiral molecules using polarization-enhanced photoacoustics
Source: Sci Adv. 2025 Mar 19;11(12):eado8012. doi: 10.1126/sciadv.ado8012 (PMC11922051; doi:10.1126/sciadv.ado8012)
Supplement: Supplementary file 1 — Supplementary Text Tables S1 and S2 Figs. S1 to S12 References [file sciadv.ado8012_sm.pdf]

Science Advances
AAAS

Supplementary Materials for

Deep tissue sensing of chiral molecules using polarization-enhanced photoacoustics

Swathi Padmanabhan and Jaya Prakash

Corresponding author: Jaya Prakash, jayap@iisc.ac.in

Sci. Adv. **11**, eado8012 (2025)
DOI: 10.1126/sciadv.ado8012

This PDF file includes:

Supplementary Text
Tables S1 and S2
Figs. S1 to S12
References

Conventional and Chiroptical Methods: *In-vivo* Feasibility Summary

A summary of the conventional and chiroptical methods highlighting various parameters like penetration depth, sample type, sample quantity and *in-vivo* feasibility in biosensing is shown in Table-S1. The various chiroptical properties that are summarised are - **CD**: Circular Dichroism, **ROA**: Raman Optical Activity, **SERS**: Surface Enhanced Raman Spectroscopy, **SEROA**: Surface Enhanced Raman Optical Activity, **SECD**: Surface Enhanced Circular Dichroism, **VCD**: Vibrational Circular Dichroism.

PAPEORS Principle

The recorded photoacoustic signal [$y(t)$] was observed to be a convolved output of the transducer impulse response [$h(t)$] and the original PA signal [$p(t)$],

$$y(t, \phi, \mu_a) = p(t, \phi, \mu_a) * h(t) \tag{S1}$$

Deconvolution of the recorded signal with the transducer impulse response was performed to determine the P and P_0 amplitudes accurately. The obtained signals were deconvolved in the frequency domain to recover the photoacoustic signal as a function of depth. The Fourier transform of the Eq. 1 results in,

$$Y(\omega) = P(\omega)H(\omega) \tag{S2}$$

The deconvolved PA signal in the frequency domain can be written as,

$$P(\omega) = \frac{Y(\omega)}{H(\omega)} \tag{S3}$$

The time-series photoacoustic data is then correlated to the depth for further extrapolation of the PA amplitudes at various depths.

$$p(t, \phi, \mu_a) = p(d, \phi, \mu_a) \tag{S4}$$

where d is the depth or path length. The detailed steps involved in extracting the points after deconvolution from $p(d, \phi, \mu_a)$ are illustrated in Fig. S1.

Table S1: Summary of Chiral Sensing techniques-I: The table summarises the parameters considered for chiral sensing like penetration depth, *in-vivo* feasibility and cost. Remarks: X* denotes that the system is under development and the method has potential for in-vivo experiments. ** for PAPEORS denotes the current parameters, which can be improved by miniaturizing the system. The chiroptical properties that are summarized are - **CD**: Circular Dichroism, **ROA**: Raman Optical Activity, **SERS**: Surface Enhanced Raman Spectroscopy, **SEROA**: Surface Enhanced Raman Optical Activity, **SECD**: Surface Enhanced Circular Dichroism, **VCD**: Vibrational Circular Dichroism.

Method	Penetration depth	In- vivo feasibility	Cost
**Chromatography**[1]	NA	X	Medium
**Electrophoresis**[1]	NA	X	Low
**Enzyme assays**[1]	NA	X	Low
**NMR Spectroscopy** [1]	NA	X	High
**Ultrasound** [2, 3]	0.1 cm	X^*	Low
**Polarimetry**[1]	Limited penetration due to multiple scattering<br>- Eye [Retina][4]: Upto 60 to 100um<br>- Skin (VIS)[5] up to 0.4 mm<br>- Skin (NIR)[6]¿300um to 1.5 mm	√	Low
**CD Spectroscopy**	NA	X	Low
**ROA Spectroscopy**	NA	X	Medium
**VCD Spectroscopy**	NA	X	Medium
**SERS/SEROA Spectroscopy**	NA^*	X^*	Low
**SECD Spectroscopy**	NA*	X^*	Low
**PAPEORS**	3.5 mm	✓	Scalable

Table S2: Summary of Chiral Sensing techniques-II: The table summarises the sample type and sample quantity required for chiral sensing. The chiroptical properties that are summarized are - **CD**: Circular Dichroism, **ROA**: Raman Optical Activity, **SERS**: Surface Enhanced Raman Spectroscopy, **SEROA**: Surface Enhanced Raman Optical Activity, **SECD**: Surface Enhanced Circular Dichroism, **VCD**: Vibrational Circular Dichroism.

Method	Sample type	Sample Quantity
**Chromatography**[1]	Tissue, Serum, plasma, urine, CSF, saliva,	10μl – 100ml
**Electrophoresis**[1]	Serum, plasma, urine, tissue	100nl – 1ml
**Enzyme assays**[1]	Serum, CSF, plasma, urine, saliva, tissue	10μl – 10ml
**NMR Spectroscopy**[1]	Urine/Fluid	10μl – 100ml
**Ultrasound** [2, 3]	Tissue*/ Blood/Fluid	2 mL
**Polarimetry**[1]	Tissue/ Blood/Fluid	10 ml
**CD Spectroscopy**	CSF, tissue/Fluids	100μl – 100ml
**ROA Spectroscopy**	Fluid	100μl – 100ml
**VCD Spectroscopy**	Fluid	100μl – 100ml
**SERS/SEROA Spectroscopy**	Fluid	10μl – 1ml
**SECD Spectroscopy**	Tissue, Urine/Fluids	10μl – 100μl
**PAPEORS**	Tissue/blood/Fluid	15 mL**

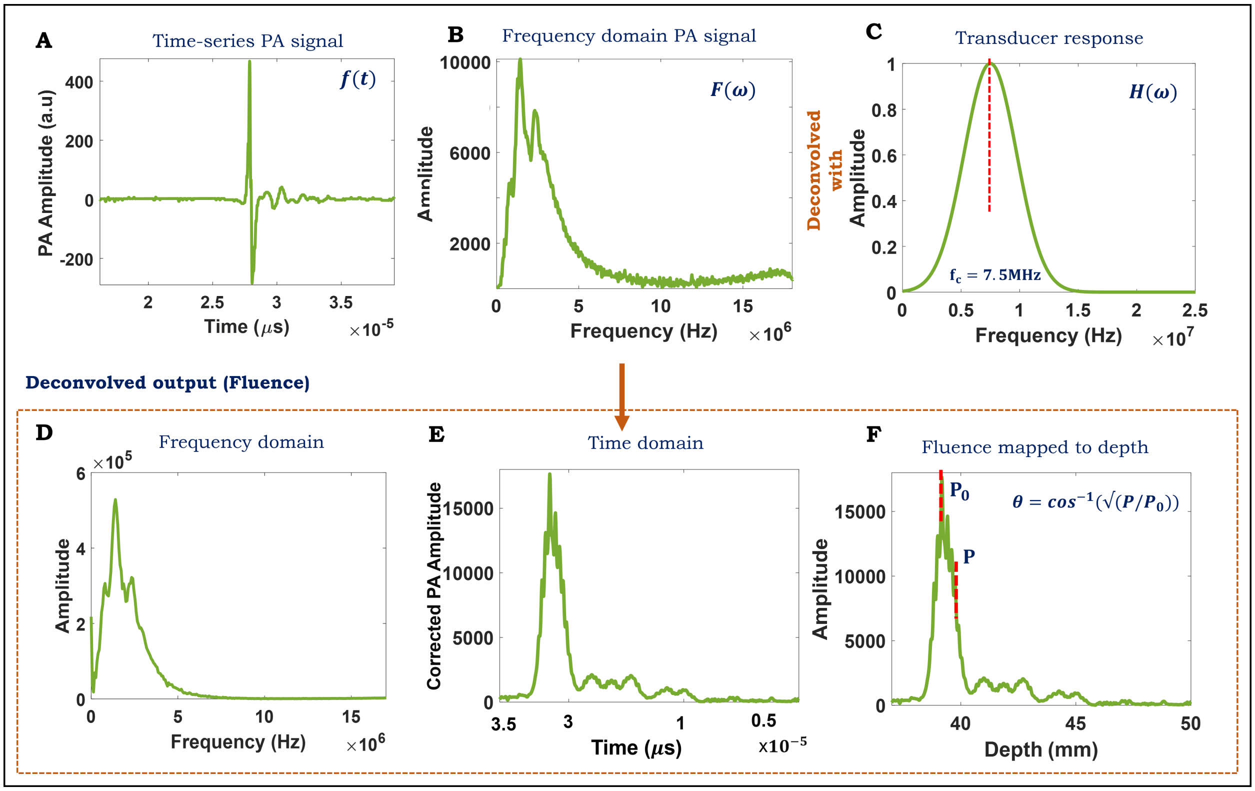

Figure S1: **Deconvolution of the time-series photoacoustic signal: (A)** shows the raw PA time-series signal, $f(t)$ from the experiment, which is a convolved output of the transducer response and the original PA signal. **(B)** is the frequency domain representation of the PA signal acquired, $F(\omega)$. **(C)** is the transducer response ($H(\omega)$) of the transducer used for the acquisition with the center frequency $f_c = 7.5MHz$. The response is reproduced from the datasheet provided by Olympus. The dotted box covering **(D)-(F)** shows the deconvolved output. **(D)** shows the frequency domain output after deconvolution of the signal with the transducer response. **(E)** represents the final deconvolved output in the time domain after taking the inverse Fourier Transform, and the response in **(E)** is mapped to depth to get the final output (fluence) that is later used for computing rotation and further calculations can be seen in **(F)**.

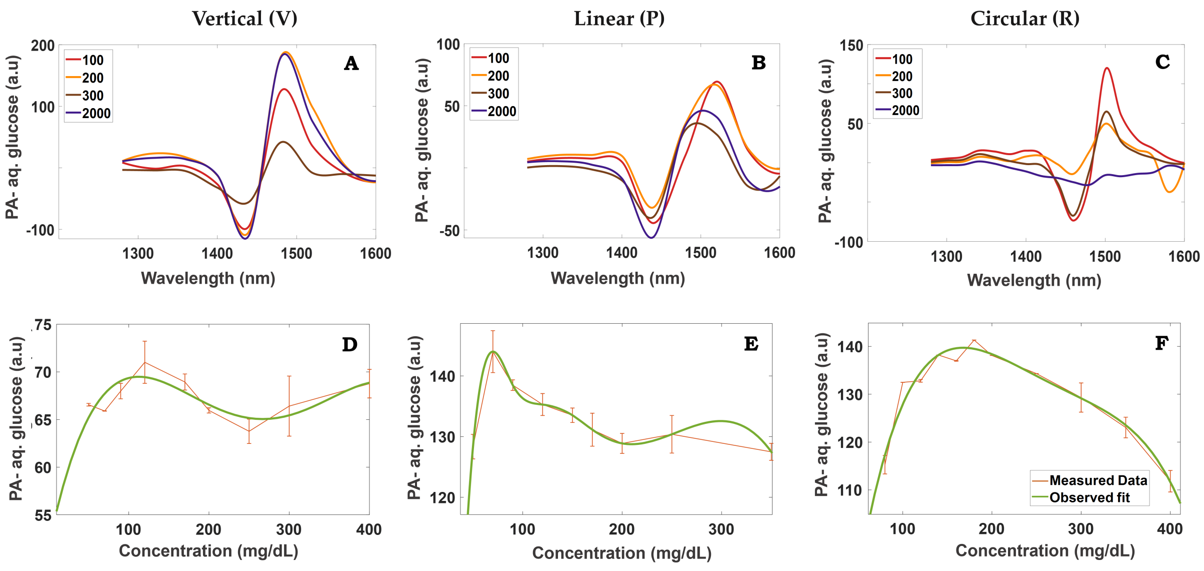

Figure S2: **Photoacoustic spectrum and non-linear effects with aqueous glucose samples** PA Spectra for **(A)** Vertical (V) incidence, **(B)** Linear (P) incidence, and **(C),** for the Circular (R) incidence. **(D), (E)** and **(F)** show the non-linear variation of the PA amplitude as a function of concentration at a depth of 1.7 mm for V, P, and R incidences, respectively.

Validation with Monte Carlo Simulations.

Polarised Monte Carlo algorithm (*48*) was used to simulate the fluence profile across the thickness of the sample considered. The near-infrared wavelengths were used for the experiment. The optical properties were similar to the experimental conditions for aqueous glucose samples were simulated to calculate the optical rotation. The absorption coefficient corresponding to different concentrations was calculated based on the molar absorptivity (*71*) at 1560 nm, was then used in the simulations. Fig. S4 shows the validation with the rotation from the experiments.

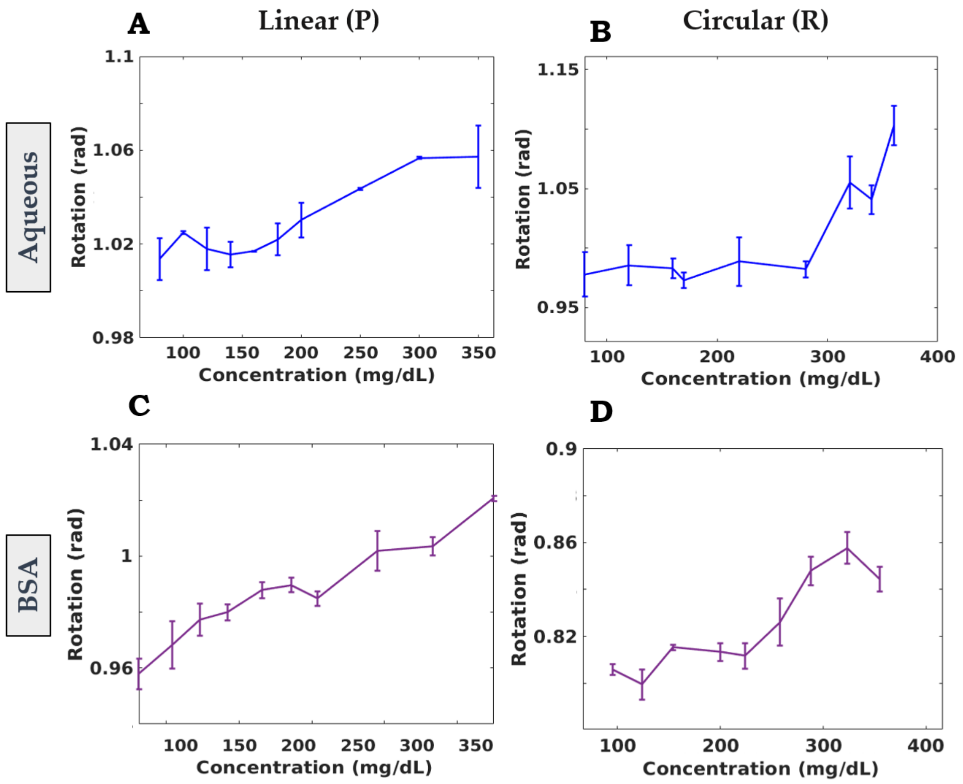

Figure S3: **Rotation from PA experiments with glucose samples: Top row**- Aqueous glucose samples and **Bottom row** BSA-based glucose samples. **(A)-(B)** represent the variation in rotation as a function of concentration at 1.7 mm for P and R polarized incidence, respectively. **(C)-(D)** represent the rotation as a function of concentration for BSA glucose samples at the same depth. The analysis showed that the variance in BSA glucose samples is lower than in the aqueous samples. The trends observed in these plots formed the basis for building the concentration estimation model. The concentrations evaluated were in the physiological range of blood glucose levels: 50-400mg/dL and BSA concentration 4g/mL of serum albumin range.

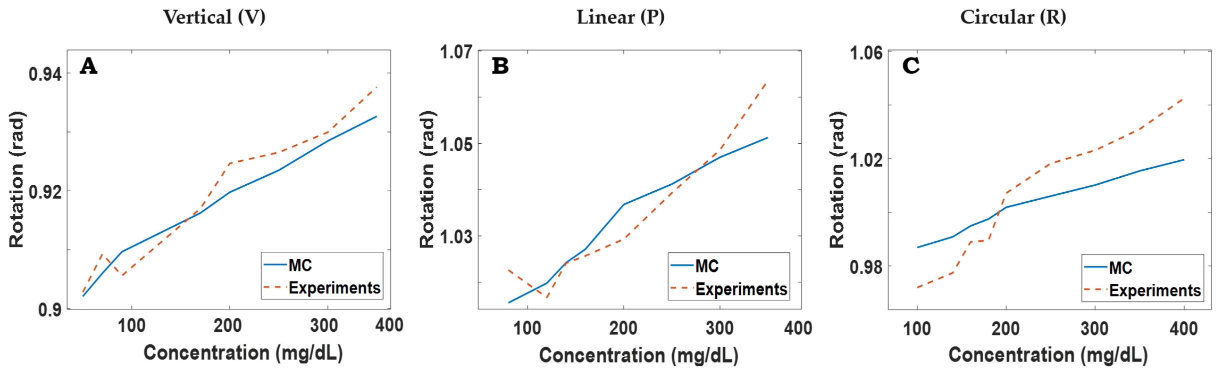

Figure S4: **Validation with Monte Carlo Simulations: (A)-(C)** shows the rotation extrapolated from the fluence profiles utilising the Polarized Monte Carlo, considering a depth of 1.7mm. The absorption coefficient corresponding to different concentrations was calculated based on the molar absorptivity at 1560nm for the simulations. The scattering coefficient was considered to be low($\mu_s = 2cm^{-1}$) considering the wavelength of incidence. The simulations were correlated with the optical rotation obtained from the fluence profiles using PA measurements. The figure shows the validation for V, P, and R incidences, respectively.

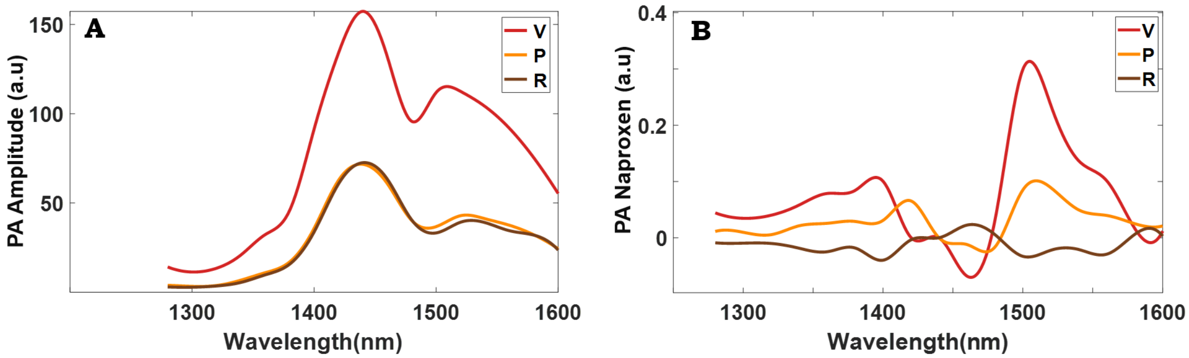

Figure S5: **Photoacoustic Spectra for Naproxen: (A)** shows the PA spectra for the Naproxen in 70% ethanol for the V, P and R incidences. The dominant absorption of water and ethanol can be observed in **(A)**. The spectra after correcting for the ethanol absorption can be seen in **(B)**. The unique absorption of Naproxen was identified to be close to 1500 nm and was further used for the rotation experiments. Changes was observed in the Naproxen spectra with mild wavelength shift in the peak absorption. V and P incidence shows a similar trend, while R incident configuration does not show a clear peak in comparison to V and P.

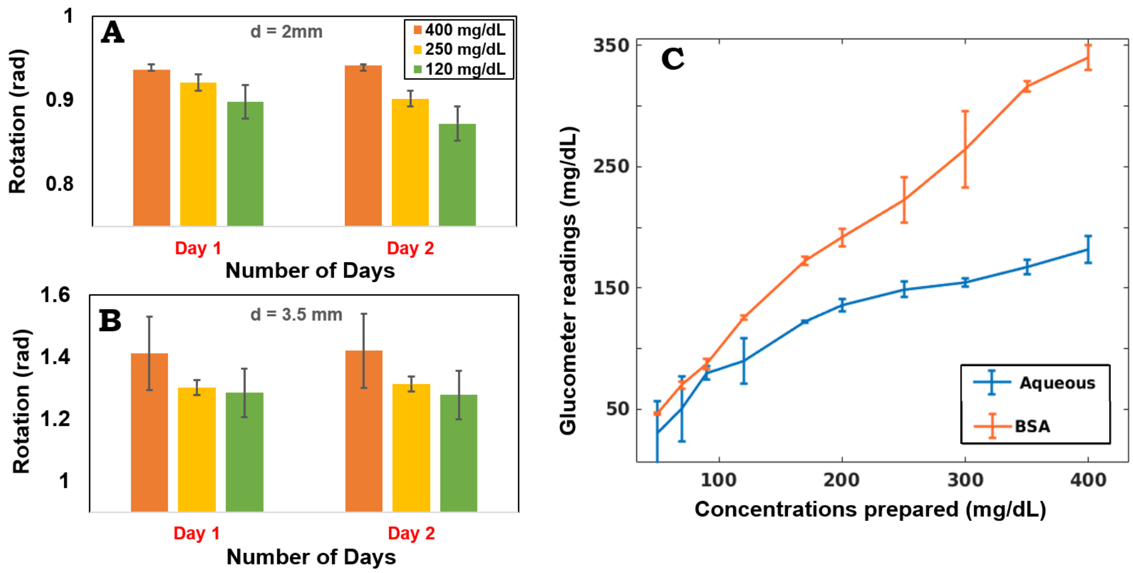

Figure S6: **Repeatability and Glucometer Validation** (**A**) and (**B**) shows the repeatability of extrapolating the magnitude of rotation from the experiments from two different days for 400 mg/dL, 250 mg/dL and 120 mg/dL for V incidence for glucose samples. (**A**) shows the data for a depth of 2mm and (**B**) for 3.5mm. The standard deviation is shown for Day 1 and Day 2 as inset. (**C**) The concentrations prepared are contrasted with the glucometer readings for the aqueous and BSA glucose samples. The deviation for the aqueous glucose samples is found to be quite high compared to the reading obtained from the BSA glucose samples. The possible reason for that could be that the albumin present in the sample makes the sample more similar to the blood plasma.

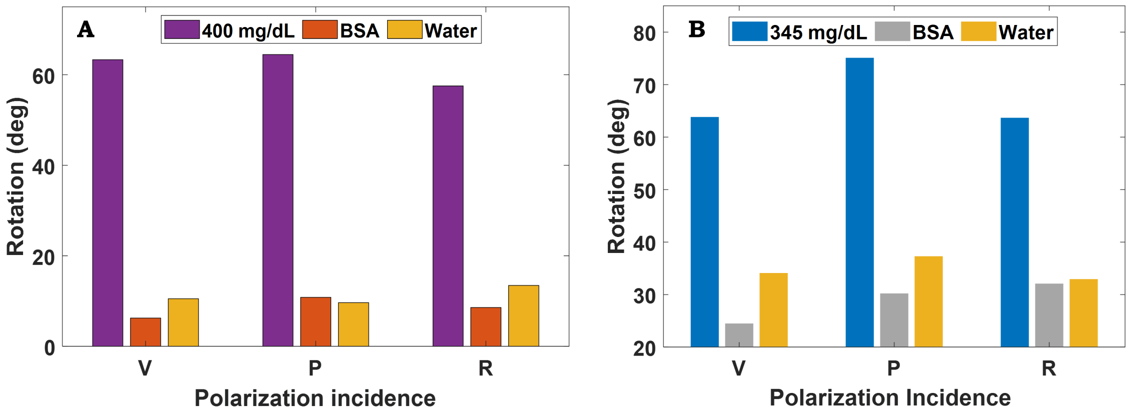

Figure S7: **Evaluation of optical rotation of glucose: (A)** The plot shows the rotation of glucose dissolved in BSA(400 mg/dL), BSA and Water without glucose at 1560nm for V, P and R polarization states, respectively. The rotation of BSA and water magnitude is negligible compared to Glucose. **(B)** shows the rotation from *ex-vivo* experiments contrasting the rotation with a control group having water as samples. The plot shows *ex-vivo* glucose sample has high rotation compared to water, magnitude is negligible. The sample with BSA shows slightly higher rotation than water due to the presence of albumin and fat from the chicken tissue slices.

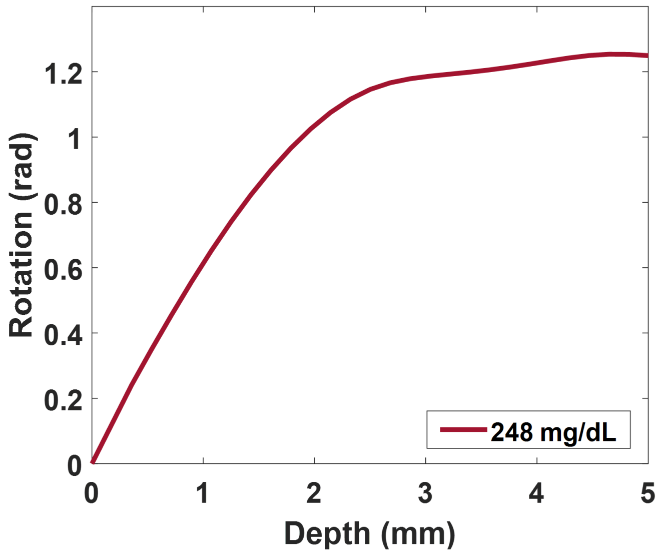

Figure S8: **Rotation as a function of depth:** The plot shows rotation varied with depth for a concentration of 248mg/dL glucose sample dissolved in BSA. The rotation angle can be seen to increase from near zero to higher magnitude as we increase the pathlength or depth considered. The curve is plotted for one concentration from Vertical incidence data to show the variations in rotation with depth. The pathlength was varied from 0 to 5mm for the analysis.

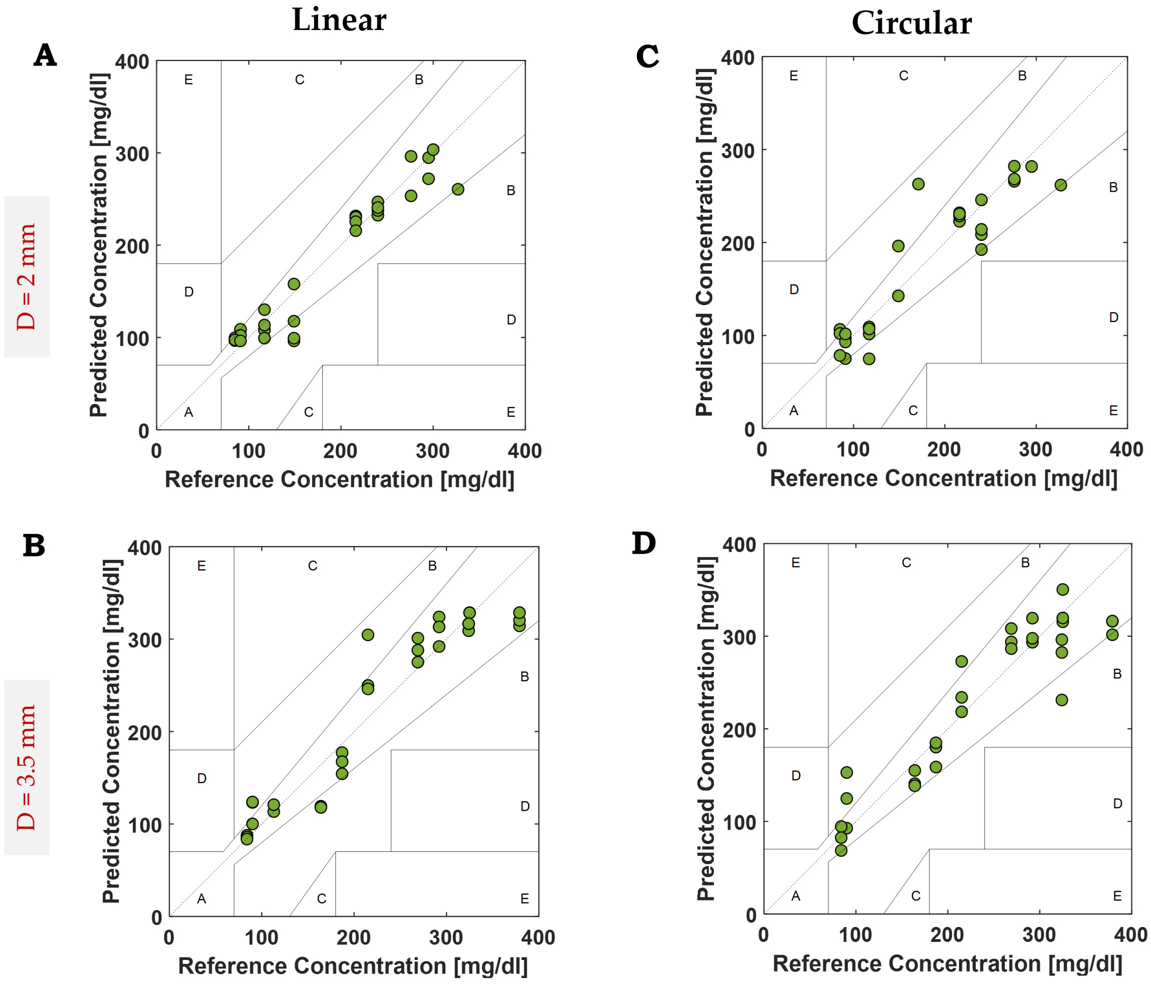

Figure S9: **CEGA for *ex-vivo* experiments with P and R incidences for the repeated set: (A)** and **(C)** in the top row shows the CEGA for a thickness of 2mm for P and R incidences respectively. **(B)** and **(D)** in the bottom row shows the CEG while the chicken slice has a thickness of 3.5mm. The number of samples was increased to 30 to better understand the estimation accuracy.

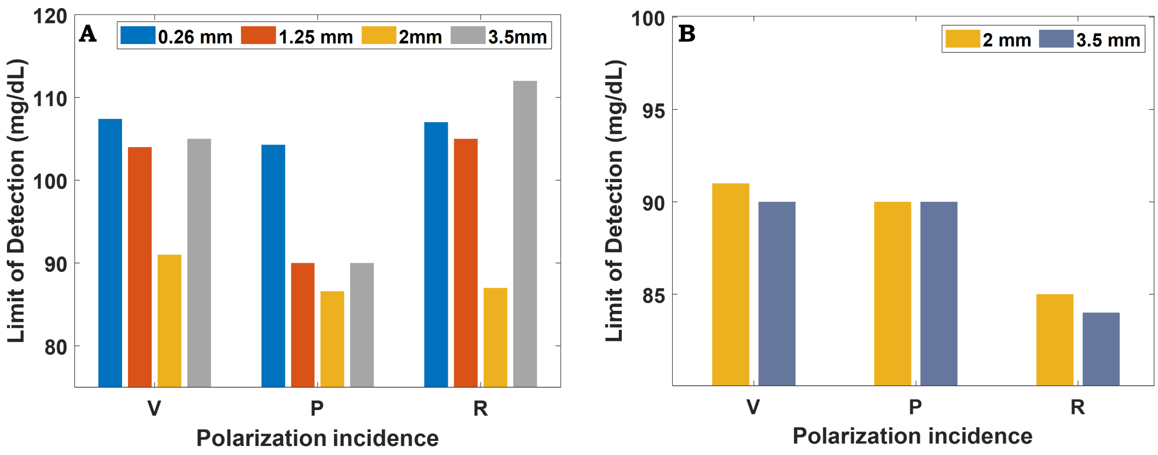

Figure S10: **Variation in LOD with depth/thickness:** The Limit of Detection variation with depth/pathlength for V, P and R incidence: **(A)** shows the LOD variation for 0.26, 1.25, 2 and 3.5mm from serum glucose samples. The detection limit is observed to decrease up to 2 mm and increase at 3.5mm. The lowest LOD is currently observed at 2mm for all the three incidences with 91 mg/dL, 86.6 mg/dL and 87 mg/dL. **(B)** shows the LOD for the 2mm and 3.5mm thicknesses from the *ex-vivo* experiments.

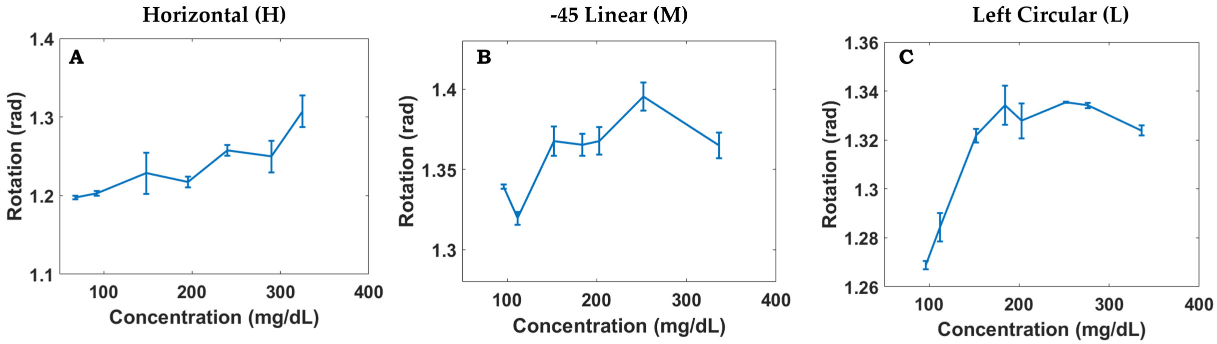

Figure S11: **Rotation from PA experiments with glucose samples for the H, M and L polarizations:** The figure shows rotation as a function of concentration for **(A)** Horizontal(H), **(B)** -45 degrees linear (M) and **(C)** left circular (L) polarization states at 1.7mm depth. The order of magnitude of rotation is slightly different for all the polarized incidences. This can be attributed to the fluence incident and the Brownian motion of the particles. The concentrations evaluated were in the physiological range of blood glucose levels: 70-400mg/dL and BSA concentration 4g/mL of serum albumin range.

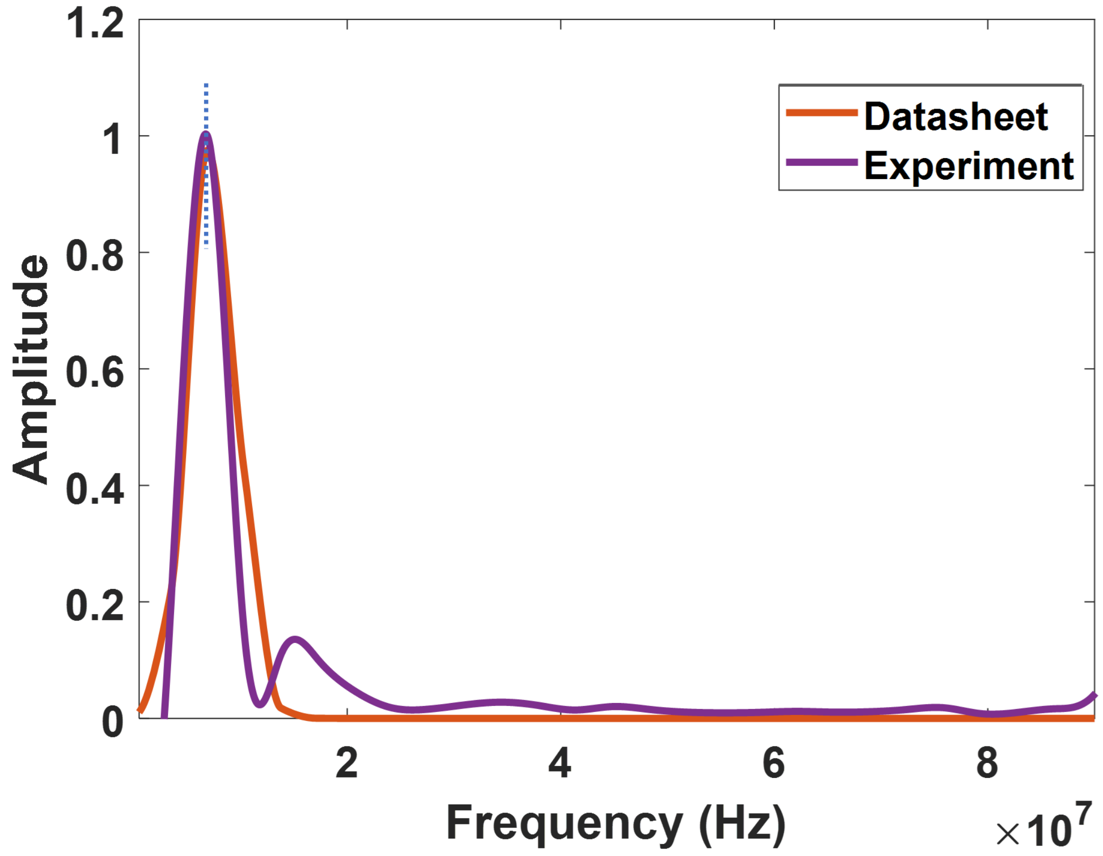

Figure S12: **Experimental characterisation of Impulse response of the transducer:** Impulse response of the transducer obtained from the experiments. The experimental response is overlaid with the manufacturer-provided impulse response. The detected center frequency (7.2MHz) matched well with the data provided by the manufacturer.

REFERENCES AND NOTES

1. J. D. Marth, A unified vision of the building blocks of life. *Nat. Cell Biol.* **10**, 1015 (2008).

2. B. K. W. Thomson, *The Molecular Tactics of a Crystal* (Good Press, 2021).

3. W. H. Brooks, W. C. Guida, K. G. Daniel, The significance of chirality in drug design and development. *Curr. Top. Med. Chem.* **11**, 760–770 (2011).

4. J. Kypr, I. Kejnovská, D. Renčiuk, M. Vorlíčková, Circular dichroism and conformational polymorphism of DNA. *Nucleic Acids Res.* **37**, 1713–1725 (2009).

5. S. M. Morrow, A. J. Bissette, S. P. Fletcher, Transmission of chirality through space and across length scales. *Nat. Nanotechnol.* **12**, 410–419 (2017).

6. J.-L. Bégin, A. Jain, A. Parks, F. Hufnagel, P. Corkum, E. Karimi, T. Brabec, R. Bhardwaj, Nonlinear helical dichroism in chiral and achiral molecules. *Nat. Photonics* **17**, 82–88 (2023).

7. N. Berova, L. Di Bari, G. Pescitelli, Application of electronic circular dichroism in configurational and conformational analysis of organic compounds. *Chem. Soc. Rev.* **36**, 914–931 (2007).

8. C. Therapontos, L. Erskine, E. R. Gardner, W. D. Figg, N. Vargesson, Thalidomide induces limb defects by preventing angiogenic outgrowth during early limb formation. *Proc. Natl. Acad. Sci. U.S.A.* **106**, 8573–8578 (2009).

9. D. M. Burley, W. Lenz, Thalidomide and congenital abnormalities. *Lancet* **279**, 271–272 (1962).

10. Y. Liu, Z. Wu, D. W. Armstrong, H. Wolosker, Y. Zheng, Detection and analysis of chiral molecules as disease biomarkers. *Nat. Rev. Chem.* **7**, 355–373 (2023).

11. L. Habartová, B. Bunganič, M. Tatarkovič, M. Zavoral, J. Vondroušová, K. Syslová, V. Setnička, Chiroptical spectroscopy and metabolomics for blood-based sensing of pancreatic cancer. *Chirality* **30**, 581–591 (2018).

12. X. Wang, J. Chen, H. Xu, Y. Fan, X. Wang, M. Zhang, Y. Liu, B. Li, J. Liu, H. Zhou, Construction of an ultrasensitive dual-mode chiral molecules sensing platform based on molecularly imprinted polymer modified bipolar electrode. *Biosens. Bioelectron.* **243**, 115759 (2024).

13. C. He, H. He, J. Chang, B. Chen, H. Ma, M. J. Booth, Polarisation optics for biomedical and clinical applications: A review. *Light Sci. Appl.* **10**, 194 (2021).

14. N. Ghosh, I. A. Vitkin, Tissue polarimetry: Concepts, challenges, applications, and outlook *J. Biomed. Opt.* **16**, 110801–110801 (2011).

15. H. R. Lee, P. Li, T. S. H. Yoo, C. Lotz, F. K. Groeber-Becker, S. Dembski, E. Garcia-Caurel, R. Ossikovski, H. Ma, T. Novikova, Digital histology with mueller microscopy: How to mitigate an impact of tissue cut thickness fluctuations. *J. Biomed. Opt.* **24**, 076004–076004 (2019).

16. J.-L. Wolfender, G. Marti, A. Thomas, S. Bertrand, Current approaches and challenges for the metabolite profiling of complex natural extracts. *J. Chromatogr. A* **1382**, 136–164 (2015).

17. C. Stark, Cesar Andres Carvajal Arrieta, R. Behroozian, B. Redmer, F. Fiedler, S. Müller, Broadband polarimetric glucose determination in protein containing media using characteristic optical rotatory dispersion. *Biomed. Opt. Express* **10**, 6340–6350 (2019).

18. D. Li, X. Chenxiang, M. Zhang, X. Wang, K. Guo, Y. Sun, J. Gao, Z. Guo, Measuring glucose concentration in a solution based on the indices of polarimetric purity. *Biomed. Opt. Express* **12**, 2447–2459 (2021).

19. B. D. Cameron, H. W. Gorde, B. Satheesan, G. L. Cote, The use of polarized laser light through the eye for noninvasive glucose monitoring. *Diabetes Technol. Ther.* **1**, 135–143 (1999).

20. S. L. Jacques, J. C. Ramella-Roman, K. Lee, Imaging skin pathology with polarized light. *J. Biomed. Opt.* **7**, 329–340 (2002).

21. J. Qi, T. Tatla, E. Nissanka-Jayasuriya, A. Y. Yuan, D. Stoyanov, D. S. Elson, Surgical polarimetric endoscopy for the detection of laryngeal cancer. *Nat. Biomed. Eng.* **7**, 971–985 (2023).

22. N. Lippok, M. Villiger, A. Albanese, Eelco FJ Meijer, K. Chung, T. P. Padera, S. N. Bhatia, Brett E, Depolarization signatures map gold nanorods within biological tissue. *Nat. Photonics* **11**, 583–588 (2017).

23. D. Co te, I. A. Vitkin, Balanced detection for low-noise precision polarimetric measurements of optically active, multiply scattering tissue phantoms. *J. Biomed. Opt.* **9**, 213–220 (2004).

24. D. Côté, I. A. Vitkin, Robust concentration determination of optically active molecules in turbid media with validated three-dimensional polarization sensitive monte carlo calculations. *Opt. Express* **13**, 148–163 (2005).

25. N. J. Greenfield, Using circular dichroism spectra to estimate protein secondary structure. *Nat. Protoc.* **1**, 2876–2890 (2006).

26. A. Micsonai, F. Wien, L. Kernya, Y.-H. Lee, Y. Goto, M. Réfrégiers, J. Kardos, Accurate secondary structure prediction and fold recognition for circular dichroism spectroscopy. *Proc. Natl. Acad. Sci.* **112**, E3095–E3103 (2015).

27. J. Kwon, K. H. Park, W. J. Choi, N. A. Kotov, J. Yeom, Chiral spectroscopy of nanostructures. *Acc. Chem. Res.* **56**, 15229–15237 (2023).

28. M. Arabi, A. Ostovan, Y. Wang, R. Mei, L. Fu, J. Li, X. Wang, L. Chen, Chiral molecular imprinting-based sers detection strategy for absolute enantiomeric discrimination. *Nat. Commun.* **13**, 5757 (2022).

29. L. Palomo, L. Favereau, K. Senthilkumar, M. S. Ępień, J. Casado, F. J. Ramírez, Simultaneous detection of circularly polarized luminescence and Raman optical activity in an organic molecular lemniscate *Angew. Chem. Int. Ed. Engl.* **61**, e202206976 (2022).

30. S. Abdali, E. W. Blanch, Surface enhanced Raman optical activity (SEROA). *Chem. Soc. Rev.* **37**, 980–992 (2008).

31. J. Haesler, I. Schindelholz, E. Riguet, C. G. Bochet, W. Hug, Absolute configuration of chirally deuterated neopentane. *Nature* **446**, 526–529 (2007).

32. V. Parchaňský, J. Kapitán, P. Bouř, Inspecting chiral molecules by Raman optical activity spectroscopy. *RSC Adv.* **4**, 57125–57136 (2014).

33. A. Taruttis, V. Ntziachristos, Advances in real-time multispectral optoacoustic imaging and its applications. *Nat. Photonics* **9**, 219–227 (2015).

34. M. Tripathi, S. Padmanabhan, J. Prakash, A. M. Raichur, Seed-mediated galvanic synthesis of CuS–Au nanohybrids for photo-theranostic applications. *ACS Appl. Nano Mater.* **6**, 14861–14875 (2023).

35. N. Liu, V. Gujrati, J. Malekzadeh-Najafabadi, J. P. F. Werner, U. Klemm, L. Tang, Z. Chen, J. Prakash, Y. Huang, A. Stiel, G. Mettenleiter, M. Aichler, A. Blutke, A. Walch, K. Kleigrewe, D. Razansky, M. Sattler, V. Ntziachristos, Croconaine-based nanoparticles enable efficient optoacoustic imaging of murine brain tumors. *Photoacoustics* **22**, 100263 (2021).

36. G. Hong, S. Diao, J. Chang, A. L. Antaris, C. Chen, B. Zhang, S. Zhao, D. N. Atochin, P. L. Huang, K. I. Andreasson, C. J. Kuo, H. Dai, Through-skull fluorescence imaging of the brain in a new near-infrared window. *Nat. Photonics* **8**, 723–730 (2014).

37. Q. Miao, K. Pu, Organic semiconducting agents for deep-tissue molecular imaging: Second near-infrared fluorescence, self-luminescence, and photoacoustics *Adv. Mater.* **30**, 1801778 (2018).

38. G. Hong, A. L. Antaris, H. Dai, Near-infrared fluorophores for biomedical imaging. *Nat. Biomed. Eng.* **1**, 0010 (2017).

39. J. Shi, T. T. W. Wong, Y. He, L. Li, R. Zhang, C. S. Yung, J. Hwang, K. Maslov, L. V. Wang, High-resolution, high-contrast mid-infrared imaging of fresh biological samples with ultraviolet-localized photoacoustic microscopy. *Nat. Photonics* **13**, 609–615 (2019).

40. L. Lin, H. He, R. Xue, Y. Zhang, Z. Wang, S. Nie, J. Ye, Direct and quantitative assessments of near-infrared light attenuation and spectroscopic detection depth in biological tissues using surface-enhanced Raman scattering. *Med-X* **1**, 9, 2023.

41. J. Prakash, M. M. Seyedebrahimi, A. Ghazaryan, J. Malekzadeh-Najafabadi, V. Gujrati, V. Ntziachristos, Short-wavelength optoacoustic spectroscopy based on water muting. *Proc. Natl. Acad. Sci. U.S.A.* **117**, 4007–4014 (2020).

42. A. Ghazaryan, S. V. Ovsepian, V. Ntziachristo, Extended near-infrared optoacoustic spectrometry for sensing physiological concentrations of glucose. *Front. Endocrinol.* **9**, 112 (2018).

43. Y. Qu, L. Li, Y. Shen, X. Wei, T. T. W. Wong, P. Hu, J. Yao, K. Maslov, L. V. Wang, Dichroism-sensitive photoacoustic computed tomography. *Optica* **5**, 495–501 (2018).

44. Y. Zhou, J. Chen, C. Liu, C. Liu, P. Lai, L. Wang, Single-shot linear dichroism optical-resolution photoacoustic microscopy. *Photoacoustics* **16**, 100148 (2019).

45. Y. Zhang, C. Glorieux, S. Yang, K. Gu, Z. Xia, R. Hou, L. Hou, X. Liu, J. Xiong, Adaptive polarization photoacoustic computed tomography for biological anisotropic tissue imaging. *Photoacoustics* **32**, 100543 (2023).

46. Z. Zhang, W. Chen, D. Cui, J. Mi, M. Gen, L. Nie, S. Yang, Y. Shi, Collagen fiber anisotropy characterization by polarized photoacoustic imaging for just-in-time quantitative evaluation of burn severity. *Photon. Res.* **11**, 817–828 (2023).

47. L. D. Barron, *Molecular Light Scattering and Optical Activity* (Cambridge Univ. Press, 2009).

48. J. C. Ramella-Roman, S. A. Prahl, S. L Jacques, Three Monte Carlo programs of polarized light transport into scattering media: Part I. *Opt. Express* **13**, 4420–4438 (2005).

49. K. Maruo, Y. Yamada, Near-infrared noninvasive blood glucose prediction without using multivariate analyses: Introduction of imaginary spectra due to scattering change in the skin. *J. Biomed. Opt.* **20**, 047003–047003 (2015).

50. Q. Ji, H. He, J. Lin, Y. Dong, D. Chen, H. Ma, D. S. Elson, Assessment of tissue polarimetric properties using stokes polarimetric imaging with circularly polarized illumination. *J. Biophotonics* **11**, e201700139 (2018).

51. M. J. O'Neil, *The Merck Index: An Encyclopedia of Chemicals, Drugs, and Biologicals* (RSC Publishing, 2013).

52. Y. Li, Y. Chen, Review of noninvasive continuous glucose monitoring in diabetics. *ACS Sensors* **8**, 3659–3679 (2023).

53. A. Karlas, N. Katsouli, N.-A. Fasoula, M. Bariotakis, N.-K. Chlis, M. Omar, H. He, D. Iakovakis, C. Schäffer, M. Kallmayer, M. Füchtenbusch, A. Ziegler, H.-H. Eckstein, L. Hadjileontiadis, V. Ntziachristos, Dermal features derived from optoacoustic tomograms via machine learning correlate microangiopathy phenotypes with diabetes stage. *Nat. Biomed. Eng.* **7**, 1667–1682 (2023).

54. N. Uluç, S. Glasl, F. Gasparin, T. Yuan, H. He, D. Jüstel, M. A. Pleitez, V. Ntziachristos, Non-invasive measurements of blood glucose levels by time-gating mid-infrared optoacoustic signals. *Nat. Metab.* **6**, 678–686 (2024).

55. E. Park, Y.-J. Lee, C. Kim, T. J. Eom, Azimuth mapping of fibrous tissue in linear dichroism-sensitive photoacoustic microscopy. *Photoacoustics* **31**, 100510 (2023).

56. Z. Zhang, Y. Shi, L. Xiang, D. Xing, Polarized photoacoustic microscopy for vectorial-absorption-based anisotropy detection. *Opt. Lett.* **43**, 5267–5270 (2018).

57. Z. Zhang, Y. Shi, Q. Shen, Z. Wang, D. Xing, S. Yang, Label free visualization of amyloid plaques in Alzheimer's disease with polarization-sensitive photoacoustic mueller matrix tomography. arXiv:2207.13271 (2022).

58. E. R. Faulds, K. M. Dungan, M. McNett, Implementation of continuous glucose monitoring in critical care: A scoping review. *Curr. Diab. Rep.* **23**, 69–87 (2023).

59. E. Hegedus, S.-J. Salvy, C. P. Wee, M. Naguib, J. K. Raymond, D. S. Fox, A. P. Vidmar, Use of continuous glucose monitoring in obesity research: A scoping review. *Obes. Res. Clin. Pract.* **15**, 431–438 (2021).

60. American Diabetes Association, Diagnosis and classification of diabetes mellitus. *Diabetes Care* **33**, S62–S69 (2010).

61. A. I. Luik, Y. N. Naboka, S. E. Mogilevich, T. O. Hushcha, N. I. Mischenko, Study of human serum albumin structure by dynamic light scattering: Two types of reactions under different pH and interaction with physiologically active compounds. *Spectrochim. Acta A Mol. Biomol. Spectrosc.* **54**, 1503–1507 (1998).

62. S. Mandal, E. Nasonova, X. L. Deán-Ben, D. Razansky, Optimal self-calibration of tomographic reconstruction parameters in whole-body small animal optoacoustic imaging. *Photoacoustics* **2**, 128–136 (2014).

63. C. Dehner, G. Zahnd, V. Ntziachristos, D. Jüstel, A deep neural network for real-time optoacoustic image reconstruction with adjustable speed of sound. *Nat. Mach. Intell.* **5**, 1130–1141 (2023).

64. H. Yang, D. Jüstel, J. Prakash, A. Karlas, A. Helfen, M. Masthoff, M. Wildgruber, V. Ntziachristos, Soft ultrasound priors in optoacoustic reconstruction: Improving clinical vascular imaging. *Photoacoustics* **19**, 100172 (2020).

65. W. J. Westerveld, M. Mahmud-Ul-Hasan, R. Shnaiderman, V. Ntziachristos, X. Rottenberg, S. Severi, V. Rochus, Sensitive, small, broadband and scalable optomechanical ultrasound sensor in silicon photonics. *Nat. Photonics* **15**, 341–345 (2021).

66. Y. Liang, W. Fu, Q. Li, X. Chen, H. Sun, L. Wang, L. Jin, W. Huang, B.-O. Guan, Optical-resolution functional gastrointestinal photoacoustic endoscopy based on optical heterodyne detection of ultrasound. *Nat. Commun.* **13**, 7604 (2022).

67. D. R. Lide, *CRC Handbook of Chemistry and Physics*, (CRC press, 2004), vol. **85**.

68. R. J. Thomas, B. A. Rockwell, W. J. Marshall, R. C. Aldrich, S. A. Zimmerman, R. J. Rockwell Jr., A procedure for laser hazard classification under the z136. 1-2000 American National Standard for safe use of lasers. *J. Laser Appl.* **14**, 57–66 (2002).

69. C. Yoon, J. Kang, S. Han, Y. Yoo, T.-K. Song, J. H. Chang, Enhancement of photoacoustic image quality by sound speed correction: Ex vivo evaluation. *Opt. Express* **20**, 3082–3090 (2012).

70. M. Shi, T. Vercauteren, W. Xia, Learning-based sound speed estimation and aberration correction for linear-array photoacoustic imaging. *Photoacoustics* **38**, 100621 (2024).

71. A. K. Amerov, J. Chen, M. A. Arnold, Molar absorptivities of glucose and other biological molecules in aqueous solutions over the first overtone and combination regions of the near-infrared spectrum. *Appl. Spectrosc.* **58**, 1195–1204 (2004).
